# Encapsulated probiotics and nanoprobiotics—Biocompatible materials, processing technologies, and applications: A review

**DOI:** 10.17305/bb.2025.13322

**Published:** 2025-12-19

**Authors:** Abrar Hussain, Warisha Alvi, Hesham R El-Seedi, Syed Abid Ali

**Affiliations:** 1 Third World Center for Science and Technology, H.E.J. Research Institute of Chemistry, International Center for Chemical and Biological Sciences (ICCBS), University of Karachi, Karachi, Pakistan; 2 Department of Chemistry, Faculty of Science, Islamic University of Madinah, Madinah, Saudi Arabia; 3 International Research Center for Food Nutrition and Safety, Jiangsu University, Zhenjiang, China

**Keywords:** Probiotics, nanoprobiotics, encapsulation, nanoencapsulation, probiotication

## Abstract

Probiotic efficacy is contingent upon delivering a sufficient number of viable cells to the site of action. However, industrial processing, storage, and gastrointestinal stresses frequently diminish survival rates below the ∼10^6^–10^7^ CFU/g or mL typically required at the time of consumption. This review aims to provide a comprehensive overview of probiotic encapsulation—particularly micro- and nanoencapsulation—as a strategy to enhance viability and facilitate timely, site-specific release. We synthesized and analyzed existing literature on key encapsulating materials, including natural polysaccharides and proteins such as alginate, chitosan, pectin, starch, casein/whey, and selected synthetic pH-responsive polymers. We also examined major encapsulation techniques, including extrusion, emulsification, spray-drying, freeze-drying, electrospinning, and coacervation, with a focus on release mechanisms and compatibility with food matrices. Overall, encapsulation consistently improved resistance to acid, bile, oxygen, heat, and dehydration, often resulting in reduced viability losses compared to free cells, enhanced storage stability, and expanded applications in functional foods and novel biomedical delivery systems. Multilayer and nanoscale systems frequently provided additional protection and targeted release in the intestinal and colonic regions. However, performance is still dependent on specific strains and matrices, and challenges persist regarding process-induced damage, premature release, sensory and textural alterations, cost and scalability, and safety and regulatory standardization, particularly for nano-enabled formats. In conclusion, encapsulated probiotics represent a promising platform; however, future advancements should focus on the development of smart, stimuli-responsive materials, scalable automated manufacturing processes, and functional validation that extends beyond viable cell counts.

## Introduction

The concept of probiotics emerged in the early 20th century as researchers examined gut microbiota and its effects on human health. Studies indicated that modifying gut flora with beneficial bacteria can enhance the well-being of host organisms and extend their lifespan. Subsequent investigations explored the immunological mechanisms underlying this phenomenon, proposing various pathways through which probiotics exert their effects [1, 2]. In the 1960s, the term “probiotics” was introduced to describe microorganisms capable of modulating the immune system and supporting host health. Since then, numerous independent researchers have investigated different dimensions of probiotics. In 2002, the World Health Organization (WHO) and the Food and Agriculture Organization (FAO) defined these microorganisms. The health benefits, biotechnological properties, and versatility of probiotics have attracted significant research interest, leading to a proliferation of literature analyzing various aspects of these beneficial microbes. In 2013, experts from the International Scientific Association for Probiotics and Prebiotics (ISAPP) reached a consensus statement to further explore the potential of probiotics. According to the ISAPP, probiotics are “live microorganisms that, when administered in adequate amounts, confer a health benefit to the host” [3, 4].

When selecting probiotic strains, factors such as safety, tolerance potential, aggregation properties, metabolite production, and mucosal surface attachment are considered. Lactic acid bacteria (LAB), including lactobacilli, *Bifidobacterium*, and enterococci, have been extensively characterized for their probiotic potential. Currently, various groups of microorganisms, including bacterial and fungal species, are recognized for their probiotic capabilities [5, 6]. Probiotics have vast applications across food, pharmaceutical, biotechnological, and medical industries, primarily based on their abilities to restore gut health, aggregate, tolerate adverse conditions, and adhere to surfaces, among other biotechnological properties. These diverse applications underscore the significant socio-economic implications of probiotics on both national and international scales, with the field experiencing an annual growth rate of 8.3% [7–9]. Emerging areas of probiotic application include disease treatment and roles in food protection and biotechnology. While the potential impact of probiotics on aging, mental health, stress, psychological disorders, and cancer is acknowledged, empirical support remains limited. Nevertheless, existing data provide promising insights into the prevention and treatment of these conditions. The debate regarding the classification of probiotics as medicinal agents continues [10].

The effectiveness of probiotics largely depends on their viability and survivability in the host or during their application [11]. For probiotic viability and efficacy, it is recommended that concentrations be expressed as log_10_ CFU per mL for liquid matrices (e.g., beverages and cultures) and log_10_ CFU per g for solid or semi-solid matrices (e.g., powders and dairy products). According to FAO/WHO and EFSA guidelines, a minimum viable count of approximately 10^6^–10^7^ CFU per g or mL at the time of consumption is required to confer health benefits. Therefore, maintaining viable populations within this range after encapsulation, processing, and storage is crucial for ensuring probiotic efficacy [12, 13].

The concentrations and viable counts of probiotics may vary depending on the specific strains and the physiological conditions of the host [14]. Unfortunately, various factors, including acidity, temperature, pressure, enzymes, and other compounds, can diminish probiotic concentrations both in the host and during industrial processes, thereby limiting their applicability across different disciplines [15]. These challenges can be addressed through various techniques designed to protect probiotics in hostile environments and maintain their viability. Encapsulation, which involves enclosing cells within a protective material, is a widely employed technique. Among the various encapsulation methods, nanoencapsulation has gained attention due to its extensive applications [16].

Recently, the potential role of nanotechnology and encapsulation has been extensively explored in microbial science. In the realm of probiotics, various encapsulation techniques are utilized to safeguard the strains of interest and maximize their benefits. While a substantial body of literature exists on probiotic encapsulation, much of it focuses on specific applications. This article aims to provide a comprehensive overview of probiotic encapsulation, emphasizing encapsulating materials (EMs), their types and properties, the diverse techniques employed, and their respective advantages and disadvantages. Additionally, this article explores the mechanisms underlying these methods and highlights their multidisciplinary applications across various fields.

### Probiotics: Selection criteria, species, applications, and limitations

The spectrum of probiotics is rapidly expanding. From strain identification to probiotic validation, and from biotechnological applications to disease treatment potential, continuous growth is evident. The emergence of new strains, comprehensive profiling of existing strains, and novel applications, including personalized medicine and diet development, has resulted in a burgeoning body of literature. For instance, over 5000 publications in the past decade have explored the medical aspects of probiotics [10]. Desired technological properties in probiotic strains include robust growth, stability, favorable sensory characteristics, extended shelf life, resilience under stress, and minimal impact on food texture [17–19]. These properties can be enhanced through advanced technologies such as genetic engineering, which improves efficiency, efficacy, and tolerance, while also enabling targeted applications [20, 21].

The selection and development of probiotics is a systematic process grounded in various criteria, including the intrinsic properties of safety, metabolite production, antimicrobial potential, and viability [22, 23]. A thorough assessment of strain virulence, resistome profiling, toxicity, and survivability is also considered during selection [24, 25]. It is important to note that probiotics are strain-dependent, meaning there is no universal criterion applicable to an entire genus or species. Moreover, not all properties are required in every strain; the relevance of these characteristics depends on the specific type and application of the probiotic, with greater diversity in properties enhancing potential applications [4, 26].

The aforementioned criteria are essential when selecting probiotic strains. In the search for candidate strains, researchers have focused significantly on LAB, known for their ability to produce lactic acid and exhibit intrinsic safety [5]. Among LAB, *Lactobacilli*, *Bifidobacterium*, and *Enterococcus* have garnered particular attention. Most LAB species are classified as Generally Recognized as Safe (GRAS) or Qualified Presumptions of Safety (QPS), with the exception of the genus *Enterococcus*, which, while not included in either category, is still utilized as a probiotic [6, 27]. In addition to LAB, other species have been identified as potential probiotics, and the concept of next-generation probiotics has emerged, aiming to uncover the probiotic potential of less-explored species [28]. Strains such as *Enterococcus faecium*, *Enterococcus faecalis*, *Akkermansia muciniphila*, *Faecalibacterium prausnitzii*, and *Bacteroides fragilis* are currently being investigated for their probiotic potential, particularly in relation to specific health conditions and diseases [29, 30].

The extensive applications of probiotics underscore their significance from both national and international socio-economic perspectives. Emerging areas of probiotic application include disease treatment, food protection, and contributions to biotechnological industries [31, 32]. Given their probiotic potential, these microorganisms are being targeted for various disease treatments and physiological processes, including aging, mental health, cancer, behavioral issues, and neurological disorders, with many strains identified for these purposes [10]. Most probiotic-based food products fall under the category of functional foods, accounting for approximately 60%–70% of the total functional food market. The practice of probiotication (the addition of probiotics to food products) is rapidly growing due to its benefits in protection and shelf-life enhancement [33, 34]. Data indicate that the global probiotic market is expanding at an annual growth rate of 8.3%, with a value of $61.1 billion in 2021, projected to reach $91.1 billion by 2026 [8, 9, 33]. The probiotic market is influenced by geographic and product-specific factors [35].

Probiotics with multifactorial spectra encompass various aspects of health, biotechnology, and other industrial domains. However, several limitations must be addressed to fully harness their benefits [26]. The intrinsic properties of probiotic strains are critical for establishing their genomic-level safety. Viability and adherence at the site of action are essential criteria for probiotics [26]. Unfortunately, factors such as the presence of acids and bases, metabolite production, antipathogenic substance secretion by other microorganisms, and various chemical substances within the host’s body adversely affect the viability and survivability of probiotics. Similarly, in biotechnological and industrial contexts, factors including pressure, temperature, heat, humidity, and associated processes can compromise their survivability [36, 37]. Therefore, protecting probiotics from hostile conditions in both the host and industrial environments is imperative. Encapsulation technology provides a solution by creating a protective barrier around probiotics using natural or artificial materials, which shields them from harsh conditions such as acidity, heat, and oxygen. This protection not only extends the shelf life of probiotics but also enhances their efficacy in functional foods and facilitates their application across diverse industries [38, 39].

### Synergism of probiotics and nanotechnology: A nanobiotechnological approach

Probiotics significantly impact various facets of life; however, their effectiveness is often limited by inactivation and reduced survivability in harsh gastrointestinal tract (GIT) conditions. This limitation adversely affects their industrial applications. The rise of nanoscience, including nanoparticles, nanocomposites, and nanohybrids, has garnered attention due to their targeted delivery capabilities, efficient properties, large surface area, and enhanced functionalities [40, 41]. Consequently, integrating nanoscience with probiotics not only addresses existing limitations but also enhances their functionalities concerning survivability, viability, protection, targeted delivery, and overall effectiveness. For instance, the development of probiotic nanofibers has been shown to improve survivability in hostile gut environments [42]. Nanotechnology’s applications in probiotics are being explored through a field known as nanobiotechnology, which focuses on enhancing probiotic functions, viability, and storage stability [43]. Particular emphasis is placed on the targeted release of probiotics, with nanobiotechnological approaches validating their role in targeted delivery systems with improved efficacy. Additionally, the use of nanocarriers and nano-EMs enhances the viability, survivability, storage duration, and transport of probiotics [44, 45].

Regulating probiotics and ensuring consumer satisfaction are critical aspects of probiotic industrial development. The advancements in nanoscience and their applications in probiotics have attracted the attention of policymakers, regulatory authorities, researchers, and industrial stakeholders to collaborate and explore the full potential of this integration. Based on the integration of probiotics and nanotechnology, various food products, including nutraceuticals, Medifoods, and fermented items, are currently making significant contributions to diverse industries [42]. As this integration emerges, ongoing research is yielding substantial progress. For example, the development of engineered nanoscale materials (ENMs) aims to enhance probiotics’ adhesion, viability, delivery systems, preservation, and targeted release [42, 45–47]. Among other promising applications, nanoprobiotics have gained attention for their potential as efficient drug carriers in targeted delivery systems [46].

### The probiotic delivery system (PDS)

The site-specific and timely release of probiotics is crucial for their functional efficacy. Probiotics primarily exert their effects upon reaching the intestine, regardless of the route of administration. However, this journey is hindered by enzymes, chemicals, and harsh gut conditions, which negatively impact probiotic efficiency [48, 49]. In the intestine, mucosal cells provide a large surface area for probiotics to attach and perform actions such as restoring gut dysbiosis, modulating the immune system, and influencing mental health through the gut-brain axis [26, 50]. Therefore, ensuring a safe journey to this site of action is essential, and encapsulation plays a vital role in protecting probiotics from these detrimental factors.

Currently, both conventional and non-conventional formulations are employed to deliver probiotics to their site of action. Technologies such as microencapsulation and nanoencapsulation are utilized to shield probiotics from harsh GIT conditions while maintaining the required viable cell counts (log 10^7^ CFU/mL). The encapsulation process involves selecting suitable EMs and methods [51, 52]. Various properties are considered during material selection and method application, including biodegradability, biocompatibility, non-toxicity, target specificity, and economic feasibility [51]. The appropriate selection of these materials can enhance probiotic applications. Encapsulated probiotics are currently utilized across the food, biotechnology, and pharmaceutical industries, where they offer greater survivability and protection for viable cells [53, 54]. Other factors, such as the size of the encapsulated probiotics, are also under consideration as these properties affect the release process, intestinal adherence, and viability. In this context, smaller-sized encapsulated cells, which possess a greater surface-to-volume ratio, demonstrate improved intestinal adherence, enhanced viability, reduced stress, and site-specific release potential [55–57].

### Strategies to protect probiotics

**Figure 1.**
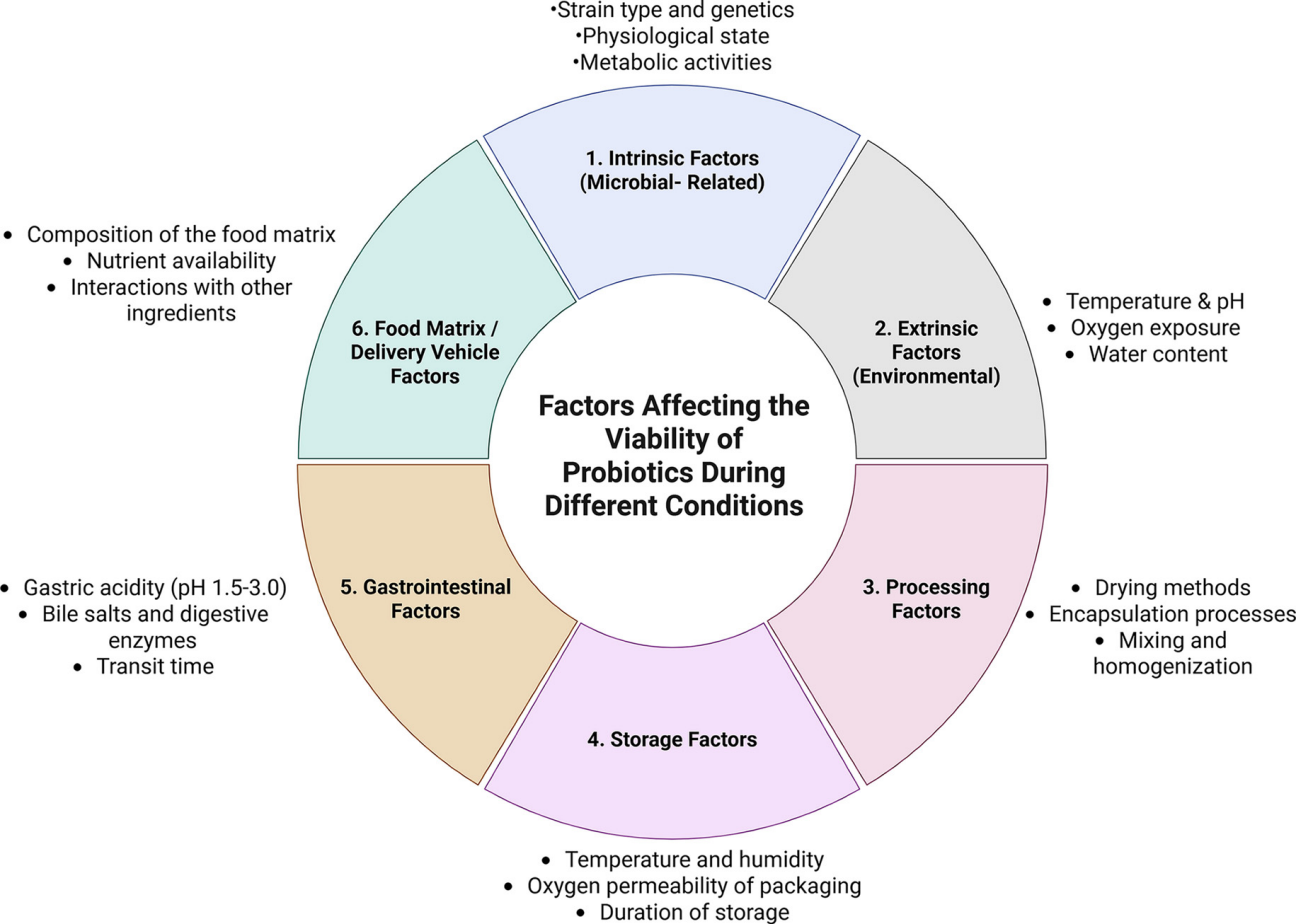
**Conceptual overview of the multidimensional factors influencing probiotic stability and viability at formulation, storage, and gastrointestinal levels.** The framework clusters key determinants into six domains—intrinsic (microbial) traits, extrinsic/environmental stressors, processing conditions, storage conditions, gastrointestinal stresses, and food-matrix/delivery-vehicle effects—highlighting their combined impact on survival during manufacturing, shelf life, and passage through the gastrointestinal tract.

The remarkable role of probiotics across various industries can be compromised by factors such as enzymatic activity, temperature fluctuations, storage conditions, and delivery methods, ultimately diminishing their market potential and therapeutic efficacy [33]. Factors affecting probiotics at different levels are summarized in Figure 1. The viability of probiotics is crucial for their effectiveness, with a requirement of more than 10^6^ CFU/g for probiotic actions [58–60]. Thus, developing cost-effective, efficient systems that bypass these obstacles and maintain probiotic viability is essential. To effectively protect probiotics, several strategies have been employed, including encapsulation, storage management, and dietary choices. Encapsulation methods such as spray drying, freeze-drying, and emulsification can shield probiotics from adverse conditions, while proper storage management, including refrigeration and avoiding direct exposure to heat or acidity, can enhance their shelf life. Additionally, incorporating prebiotic-rich foods, avoiding antibiotics, and managing stress can also support probiotic viability [61]. Emulsion, spray drying, and extrusion are commonly utilized technologies at both analytical and scalable levels. However, alternative technologies such as complex coacervation and vibrational extrusion are also yielding promising results in terms of efficiency, enhanced morphology, and functional properties [34, 57].

### Encapsulation: A promising strategy for probiotic protection

Encapsulation techniques are among the most recommended and actively utilized methods for the protection of probiotics. Within the coating material—also referred to as the carrier, membrane, or matrix—substances are classified as active, filling, or internal materials. Probiotic encapsulation can be performed using various methods, including emulsion, spray drying, extrusion, and electrospinning, which are widely employed at both analytical and scalable levels [34, 57]. Encapsulation can manifest as a reservoir or microcapsule (where active materials are enclosed), a coated matrix (where active materials are coated with protective materials), or a matrix (where active materials are dispersed within the carrier materials) [56, 60].

This technique involves covering entire bacterial cells with various materials that provide protection and enhance their properties [60, 62]. The effectiveness of probiotics can be improved by ensuring their targeted delivery to the colon, a process that can be achieved through encapsulation techniques [33, 58, 60]. Encapsulation safeguards probiotics from various environmental, industrial, and host conditions. Specifically, within the body, probiotics are released from their encapsulated shell through mechanisms such as changes in pH, temperature, solubility, rupture, biodegradation, and diffusion [59, 62]. The advantages of encapsulation include enhanced protection, contamination prevention, stability, and improved survivability [62–64].

**Figure 2.**
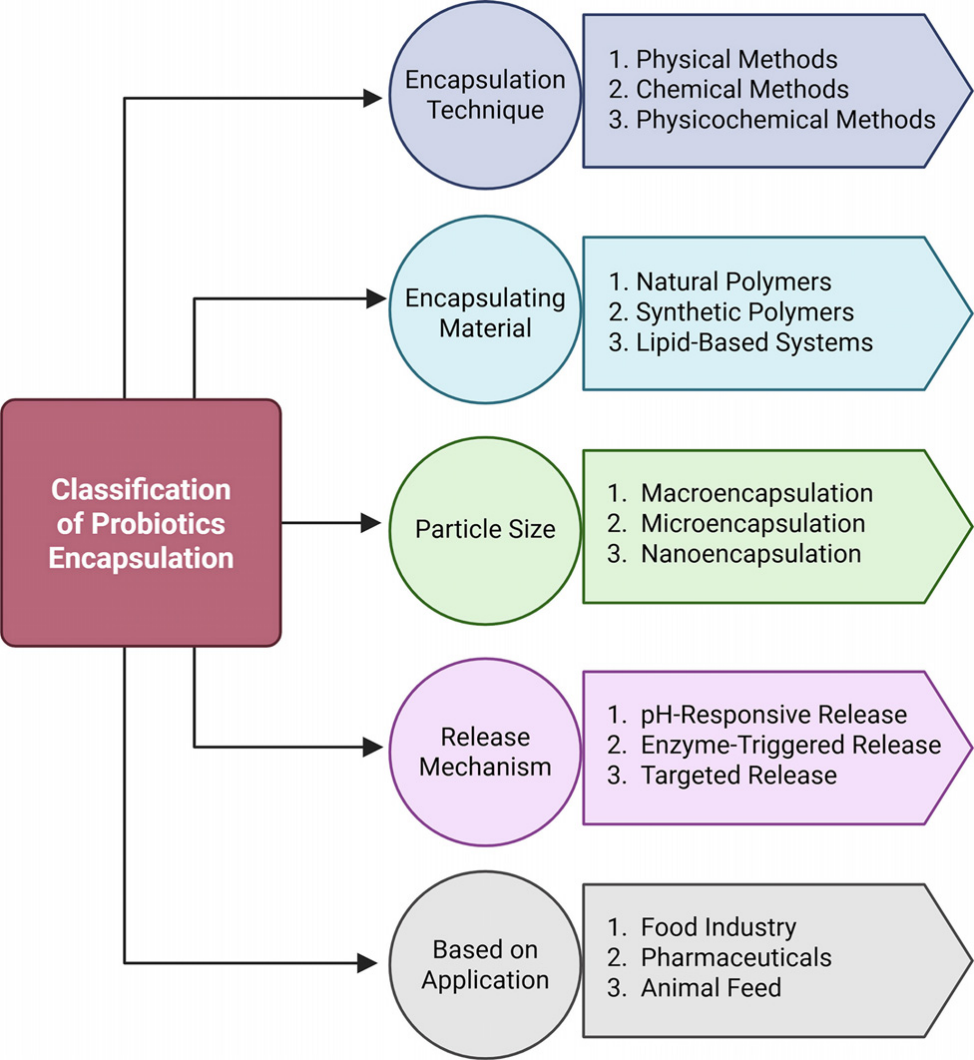
**Multi-criteria classification framework for probiotic encapsulation systems.** A given formulation can be specified by selecting options across independent design axes, including encapsulation technique, carrier material class, particle size range, release trigger, and intended application.

While encapsulation offers advanced properties over methods such as immobilization, it also presents certain limitations. Challenges in probiotic encapsulation include maintaining cell viability, integrity, the ability to withstand external pressures, and ensuring no alteration of food properties [62, 63]. Additionally, although encapsulation methods possess several advantages, they also face limitations. For example, the application of heat during spray drying may reduce probiotic viability, along with issues related to cost and time. Similarly, freeze-drying can result in ice crystal formation, potentially damaging probiotic cell membranes and reducing viability [33]. Encapsulation technologies can be classified based on methods (physical or chemical), materials (natural or synthetic), particle size (micro, meso, and nano), release mechanisms (pH-responsive, enzyme-responsive, etc.), and applications (food, animals, pharmaceuticals, etc.). A comprehensive classification system for probiotic encapsulation is summarized in Figure 2.

### Nanoencapsulation and probiotics: Nanoprobiotics

Nanoencapsulation is a technique that involves enclosing a substance within a nanoscale shell or matrix (1–1000 nm) to protect it and regulate its release. This process enhances the stability and delivery of active compounds, such as probiotics, vitamins, antioxidants, and drugs, across various products, including food, pharmaceuticals, and cosmetics. In the context of probiotics, nanoencapsulation protects cells from environmental stresses such as heat, oxygen, acidic pH, and bile salts, thereby improving their survivability during processing, storage, and gastrointestinal transit. Commonly used nanocarriers for this purpose include liposomes, nanoemulsions, solid lipid nanoparticles, polymeric nanoparticles, and nanofibers. These nanostructures provide a high surface area-to-volume ratio, facilitating more efficient encapsulation and controlled release of probiotics at targeted sites, such as the intestine or colon [42, 43].

**Figure 3.**
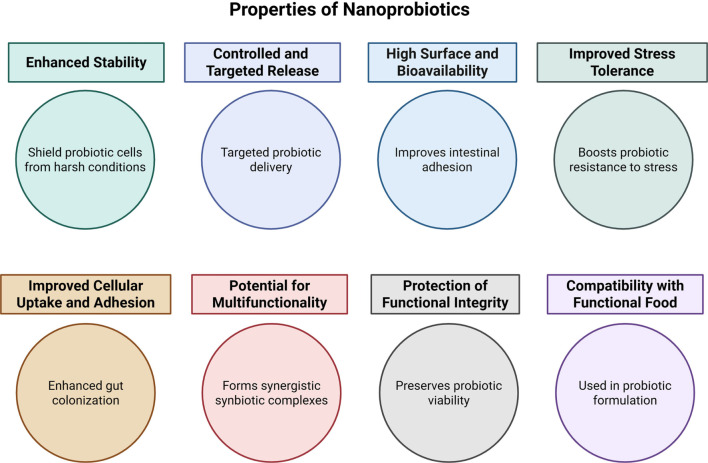
**Key attributes of nanoprobiotic formulations enabled by nanoscale carriers.** The diagram summarizes how nanoencapsulation can improve probiotic performance by enhancing stability and stress tolerance during processing/storage, increasing effective surface area to support bioavailability and intestinal adhesion, and enabling controlled or targeted release, while preserving functional integrity and allowing multifunctional designs (e.g., synbiotic co-formulations) compatible with food and biomedical applications.

The emergence of nanotechnology has opened new avenues in probiotic research and application. The integration of nanoscience with biotechnology, known as nanobiotechnology, has led to the development of nanoprobiotics, wherein probiotics or their bioactive metabolites are encapsulated, protected, or delivered using nanoscale materials. Nanoprobiotic formulations aim to address traditional challenges in probiotic delivery, including low viability during processing, reduced stability during storage, and poor survival through gastrointestinal transit. Various nanocarriers, including lipid-based nanoparticles, polymeric nanoparticles, nanofibers, and nanoemulsions, have been investigated to enhance probiotic encapsulation efficiency, targeted delivery, and controlled release. These nanosystems not only shield probiotic cells from environmental and physiological stresses but also enable site-specific delivery to the intestine, thereby improving colonization and therapeutic efficacy. Furthermore, nanoscale encapsulation of probiotic-derived postbiotics—such as bacteriocins, exopolysaccharides, and enzymes—enhances their bioavailability and functional performance in food and biomedical applications. Despite their promise, nanoprobiotic approaches face challenges related to safety assessment, large-scale production, and regulatory approval. Nonetheless, emerging evidence suggests that nanotechnology-driven strategies could revolutionize the next generation of probiotic-based functional foods and therapeutic formulations. The key properties of nanoprobiotics are summarized in Figure 3.

### Microencapsulation: Advantages and disadvantages

Microencapsulation is a process in which small amounts of active ingredients are tightly encased within a micrometer-sized shell or capsule. This small size not only protects the enclosed materials but also reduces their interaction with environmental factors [56, 59, 64]. Microencapsulation aids in sustaining probiotic viability under extreme gastrointestinal conditions, as probiotics are sealed within microcapsules. The release of probiotics from these capsules depends on specific properties of the release sites [47, 58, 65]. The shape and size of a microcapsule are determined by the material types used. Typically, the core contains liquid (with probiotics), while the exterior comprises a robust and thin membrane that protects the inner material and allows for material exchange [64]. At target sites, the microcapsule-bound cells are released due to changes in pH, pressure, and solvent conditions [64].

This technology is employed to protect probiotics and maintain their viability during GIT, storage, processing, production, and application [34, 35, 64]. Microencapsulated probiotics generally exhibit greater survival under harsh conditions compared to non-encapsulated cells; for instance, one study reported a 3–4 log cycle reduction in encapsulated cell count vs a 6–8 log cycle reduction in free cells [66]. The microencapsulation of probiotics offers several advantages, including enhanced survival, stability against adverse conditions, controlled release, and versatile physicochemical properties [35, 59]. While its protective potential is promising, certain limitations can hinder its broader application. These limitations range from the selection of suitable EMs to challenges in controlling onsite release within the gut. Additionally, the cost and complexity of the process present barriers to widespread acceptance [52].

**Figure 4.**
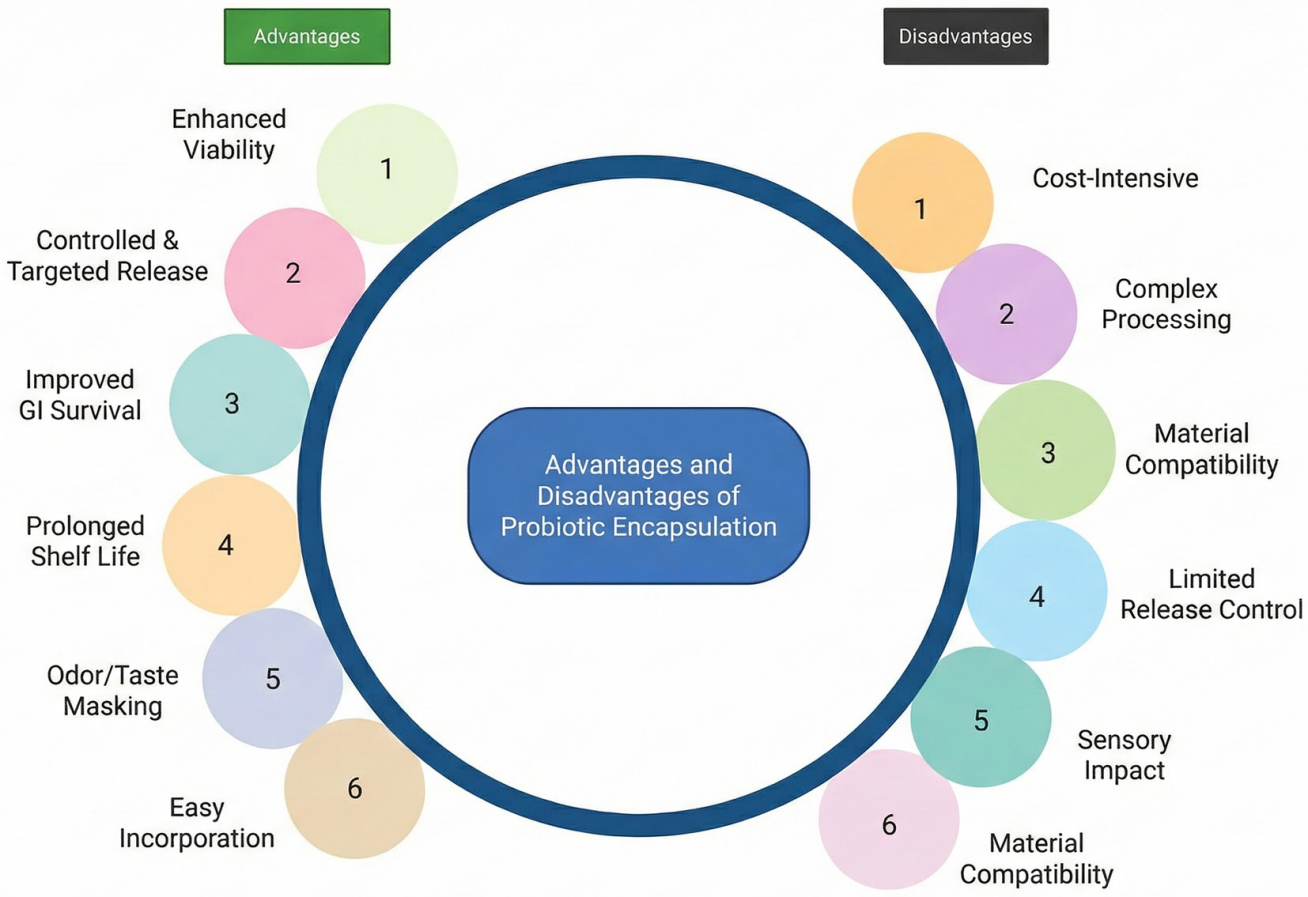
**Overview of the principal trade-offs associated with probiotic encapsulation.** The schematic contrasts commonly reported benefits relevant to maintaining viable cells through processing, storage, and gastrointestinal transit, with implementation constraints that influence translation to commercial products, including cost and process complexity, material–matrix compatibility and sensory effects, and challenges in achieving reproducible dose and release control during scale-up.

Scalability is a critical consideration in probiotic encapsulation that must be addressed during commercialization. It remains a key factor influencing the practical application and industrial adoption of encapsulation technologies. Similarly, dose optimization and the risk of early release of encapsulated cells pose additional challenges in probiotic encapsulation [67]. The major advantages and disadvantages of probiotic encapsulation are summarized in Figure 4. Another approach to encapsulating probiotics involves the use of nanomaterials, known as nanoencapsulation. This process provides protection for probiotics during their journey within the body and can enhance their storage and shelf life [63, 68, 69]. Advantages associated with this approach include high viability, greater stability, and controlled release; however, challenges such as cost, limited efficacy, potential toxicity, and complex formation also exist [63, 70, 71].

**Figure 5.**
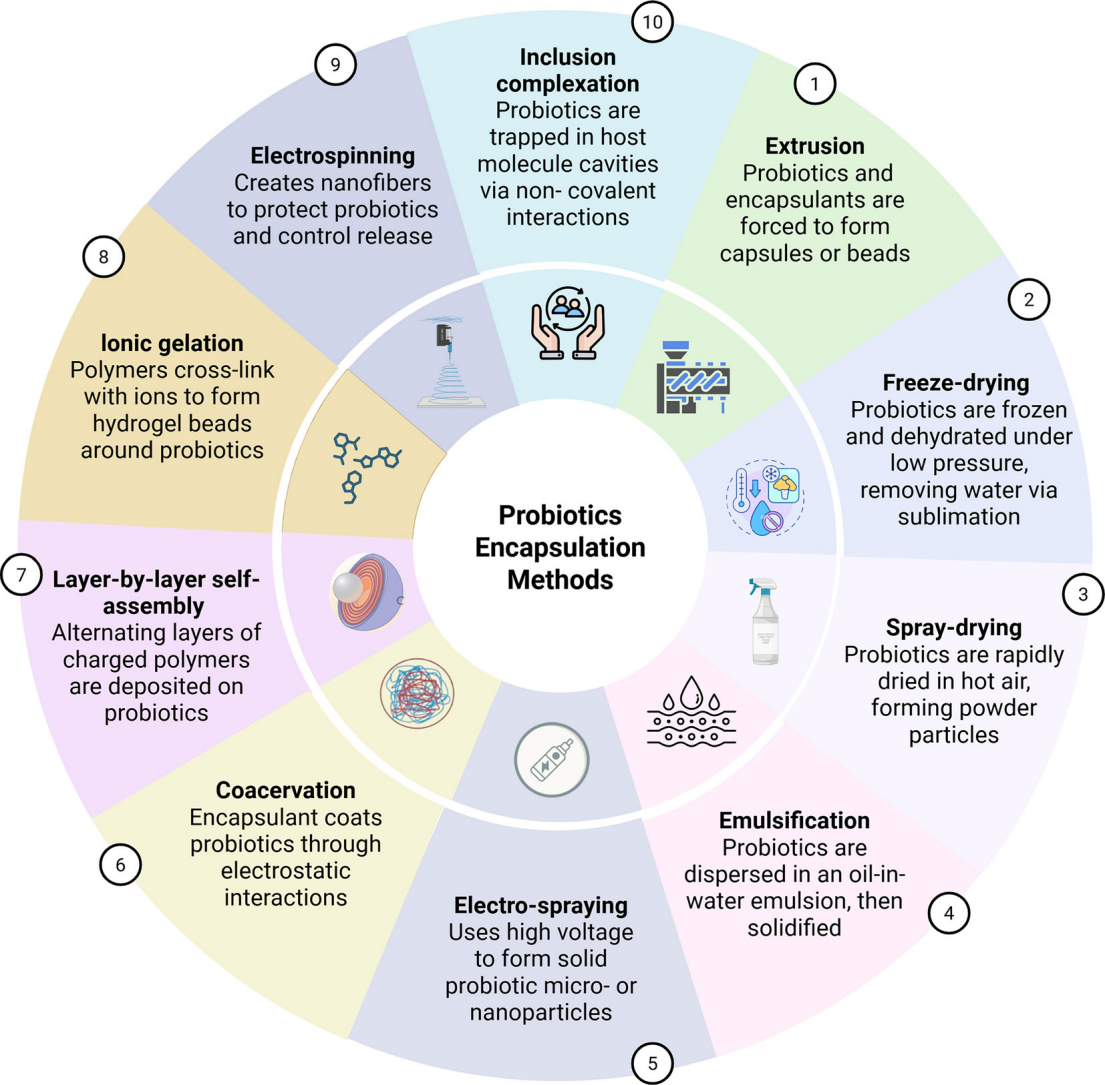
**Schematic overview of widely used probiotic encapsulation methods and their defining process principles.** Techniques are grouped by how the protective structure is formed—via droplet/bead generation and gelation (e.g., extrusion, ionic gelation), dehydration-based particle formation (freeze-drying, spray-drying), interfacial structuring (emulsification, coacervation, layer-by-layer assembly, inclusion complexation), and electrically driven fiber/particle fabrication (electrospinning, electro-spraying)—providing a common reference for selecting approaches based on practical constraints such as gentleness, cost, and scalability.

## Encapsulated methods and technologies

A variety of encapsulation techniques, numbering over a dozen, are currently employed to protect probiotics under gut conditions, during industrial processes, and throughout storage. The choice of encapsulation technique is contingent upon the specific application being studied. Generally, methods that are non-destructive, cost-effective, biologically compatible, and environmentally friendly are prioritized for probiotic encapsulation. A brief overview of various probiotic encapsulation methods is presented in Figure 5. While the range of encapsulation techniques is extensive and summarized in Table 1, this section will briefly discuss the most common methods.

### Electrospinning

Electrospinning is an encapsulation technique that utilizes a polymer solution subjected to an electric field, resulting in the formation of micro- or nanofibers. The application of the electric field induces a contraction in the surface tension of the polymer droplet, transforming it into a jet that elongates and thins. Concurrently, the solvent evaporates, solidifying and depositing the polymer onto a collector [15, 64]. This technique is advantageous due to its reduced reliance on solvents and lower temperature requirements, making it widely applicable in drug delivery, food processing, biotechnology, and biomedicine [72]. Additional benefits include simplicity, versatility, minimal thermal damage, and scalability [60].

Electrospinning is also utilized for probiotic encapsulation, wherein probiotics are integrated into the fibers, allowing them to remain viable for extended periods. The high surface-to-volume ratio and rapid dissolution potential of the fibers facilitate targeted release of the probiotics [64]. Several studies have employed electrospinning for probiotic encapsulation with promising results. For instance, Wei et al. [73] encapsulated *L. plantarum* using synthetic polyvinyl alcohol (PVA) and polyvinyl alcohol/silk fibroin (PVA/SF) nanofibers, observing greater viability compared to free cells after treatment with gastric juice for 2 h. Another study encapsulated various *Lactobacillus* strains using a blend of gum Arabic and pullulan via electrospinning, demonstrating enhanced survivability and high viability during four weeks of storage at 4 ^∘^C [74].

Electrospinning techniques can be categorized into five types: blend electrospinning, coaxial electrospinning, melt electrospinning, gas jet electrospinning, and emulsion electrospinning [64]. However, blend and coaxial electrospinning are primarily utilized for probiotic encapsulation.

### Blend electrospinning

Blend electrospinning is a prevalent method in which probiotics are dispersed within a polymer solution. The incorporation of probiotics alters the viscosity and conductivity of the dispersion. Studies utilizing scanning electron microscopy have revealed variations in fiber morphology, indicating differences in thickness between fibers containing probiotics and those without. The morphology and hydrophobic properties of the cells significantly influence fiber formation. Overall, this method enhances probiotic stability during storage [64]. For example, encapsulation of *L. acidophilus*B1075 via blend electrospinning exhibited greater survivability compared to other methods such as freeze-drying and spray-drying. The rapid release characteristics of the blend encapsulation method are particularly advantageous for drug delivery systems [64].

### Coaxial electrospinning

Coaxial electrospinning is a widely utilized technique in biological applications that produces core-shell nanofibers when the solution is extruded from a nozzle. In this process, active cells, such as probiotics, are encapsulated within the core, while the outer shell provides protection, thus preserving higher cell viability and enhancing oxidative stability [64]. For instance, Yang et al. [75] demonstrated that electrospun core-shell fibers encapsulating fish oil extended shelf life by 65 days compared to non-encapsulated oils. Similarly, *L. paracasei* was encapsulated in starch-formate/glycerol fibers using coaxial electrospinning, maintaining viability for 21 days when stored at 4 ^∘^C [76].

### Emulsification

Emulsification involves the combination of two immiscible liquids, such as oil and water, into a stable and uniform mixture known as an emulsion, facilitated by hydrophilic and hydrophobic substances [77]. This method is employed to encapsulate various bioactive substances and finds applications across food, pharmaceutical, and cosmetic industries [78]. The two-phase Pickering emulsion consists of a continuous phase (forming the emulsion) and a discontinuous phase (containing the cells). This advanced and highly compatible technique is particularly effective for encapsulating probiotics [79]. Initially, an emulsion is formed by dispersing an aqueous alginate-probiotic mixture into an oil phase. Subsequently, calcium chloride (CaCl_2_) is added to induce ionic crosslinking of alginate, converting the dispersed droplets into gelled microbeads. Finally, the oil phase is removed to recover the encapsulated probiotic beads [60, 70].

Probiotic encapsulation via emulsification allows for the production of uniformly sized cells (achieved by controlling agitation speed and water–oil ratios), a higher survival rate, and large-scale industrial production [60]. Although widely employed in the food industry, the emulsification method has the limitation of necessitating additional materials, which may be considered undesirable [60]. da Silva et al. (2023) recently encapsulated *L. acidophilus* NRRL B-4495 and *Lactiplantibacillus plantarum* NRRL B-4496 using alginate and gelatin-based microparticles through emulsification. Their findings revealed higher survivability for up to 120 days at both 5 ^∘^C and 25 ^∘^C [80]. However, while this method effectively preserves probiotic viability during storage, the inclusion of non-food-grade or excessive stabilizing agents may compromise the natural quality and consumer perception of the final product.

### Freeze-drying or lyophilization

The freeze-drying method is predicated on the removal of water content and moisture from frozen products under vacuum conditions, utilizing the phenomenon of sublimation. Freeze-drying has applications across various industries and aids in maintaining the textural properties of substances [81]. Subjecting viable cells, such as probiotics, to low temperatures can enhance their storage life and viability, making it a crucial method for thermal-sensitive probiotics [35, 64, 70]. The general mechanism of freeze-drying consists of three stages: freezing, primary drying, and secondary drying. In the first stage, materials are frozen at very low temperatures, followed by water removal under vacuum in the second stage. The secondary drying phase involves the removal of unfrozen water [60, 64, 70].

**Table 1:** Overview of various probiotic encapsulation methods, including procedural steps, advantages, and limitations

**Methods**	**Mechanism**	**Advantages**	**Disadvantages**	**Applications**	**References**
Spray-drying	Atomization, mixing, separation	It is a cheap, easy, and scalable method that gives high efficiency, viability, and stability and reduces moisture content.	It is time-consuming and decreases the viability of probiotics. It also causes thermal cell inactivation.	Food, biotechnology, pharmaceuticals, and chemical industries.	[33, 56, 60, 64]
Freeze-drying	Freezing, sublimation, desorption	Improve viability, enhance stability, and have minimum destruction.	It is costly and can cause damage to probiotics due to the formation of ice crystals.	Food, biotechnology, pharmaceuticals, and heat-sensitive materials industries.	[33, 56, 64]
Extrusion	Mixing, droplet formation, gelation, hardening	It is a simple, cheap, and scalable technique (at pilot scale) that enhances the viability and stability.	The size is large and has issues in scalability (at industrial scale) and humidity control.	Food, biotechnology, pharmaceutical, and feed industries.	[33, 56, 57]
Emulsification	Mixing, emulsion, gelation, solidification	It enhances the viability and protects it from harsh conditions with controlled over size (under optimized conditions) and targeted release.	The morphology is not in control and requires an additional layer for protection.	Food, pharmaceutical, cosmetics, dyeing, and plastic industries.	[33, 60, 77, 89]
Electrospraying	Atomization, droplet formation, deposition, evaporation	It gives monodisperse particles with control over size. It enhances stability and viability.	It has a lower production rate, and an external force is applied, which affects the functions.	Food, biotechnology, pharmaceuticals, and therapeutics.	[60, 90]
Coacervation	Mixing, coacervation, microcapsules	It has the properties of versatility, easy operation, biocompatibility, and inexpensiveness.	It has a complex process, needs a specific pH and ionic strength, and has a lower release potential.	Biotechnology, agriculture, textiles, pharmaceuticals, and food industries.	[40, 88]
Layer-by-layer (LbL) self-assembly	Preparation, deposition, attraction, stabilization	It enhances the probiotic viability, shows resistance to harsh conditions, and improves the adhesion.	It is a time-consuming process, and the membrane may disrupt, thus requiring more layers.	Food, biotechnology, pharmaceuticals, cosmetics, and animal feed industries.	[40, 91, 92]
Fluidized bed coating	Coating, spraying, evaporation	Uniform layers are formed and protect the probiotics from harsh conditions.	It is expensive and has exposure to high temperature.	Food, bakery, biotechnology, and meat industries.	[70, 93]
Ionic Gelation	Electrostatic attraction, stirring, ionic gelation, drying	It is an easy, inexpensive, and fast method that does not require an organic solvent.	It has difficulty in uniform-size particle formation.	Food, pharmaceuticals, biomedical, and other industries.	[70, 94]
Inclusion Complexation	Interaction, penetration, entering cavity	It has controlled release, enhanced shelf life, and stability.	It only uses cyclodextrin, requires high energy, and has low water solubility.	Food, pharmaceuticals, biomedical, and other industries.	[70, 95]
Spray cooling	Atomization, cooling, solidification.	It has low cost, fast processing, and no need for organic solvents.	It needs high operation temperature, low encapsulation efficiency, and low viability.	Food, biotechnology, and cosmetics industries.	[70, 96]
Electrospinning	Preparation, electric field application, jet formation, stretching and evaporation	Electrospinning creates porous nanofibers that protect probiotics, enable controlled release, and preserve viability at mild temperatures.	Electrospinning faces challenges such as low encapsulation efficiency for hydrophilic bacteria, potential electric or solvent stress on cells, and difficult, time-consuming scale-up.	Film coatings, protective matrices, scaffold-like structures for probiotics.	[73, 90]

Although this method is widely adopted across industries, it does present certain disadvantages, including high costs, extended processing times, ice crystal formation, and challenging water removal, which can negatively impact probiotic viability and survivability [35, 56, 64]. This method is best suited for probiotics that are tolerant to low temperatures, necessitating a preliminary tolerance assessment to ensure the probiotics can withstand low-temperature conditions [60]. Studies utilizing freeze-drying for probiotic encapsulation have reported higher viability, improved storage capacity, and extended shelf life. For instance, a study encapsulating *L. acidophilus* with a blend of pectin microparticles via freeze-drying found enhanced probiotic viability for four months at 25 ^∘^C [82]. In another study, two probiotic strains, *L. acidophilus* and *L. casei*, were encapsulated using whey protein isolates and fructooligosaccharides as encapsulating agents, demonstrating higher survivability and greater tolerance to gastric conditions after one month of storage at 4 ^∘^C [83].

### Spray drying

Spray drying is a technique that rapidly transforms a liquid into a dry powder by atomizing it into fine droplets and exposing these droplets to a hot drying medium, facilitating the instant evaporation of the solvent. The resultant dry powder particles are subsequently separated from the gas and collected [15, 60, 70]. This method offers several advantages, including low cost, multifunctionality, continuous operation, easy accessibility, and improved storage stability [56]. However, it also presents certain disadvantages, such as thermal stress, dehydration, osmotic pressure, and oxidative stress, which can inactivate probiotics and reduce their viability [39, 57, 64]. To mitigate these challenges, various strategies, such as the use of cryoprotectants and optimization of inlet and outlet temperatures, are implemented. Incorporating protectants like starch and fiber can further enhance probiotic viability during storage [64]. Spray drying typically yields microcapsules ranging from 10 to 150 µm in size, large enough to encapsulate multiple probiotic cells within a single particle, thereby improving their protection and stability [60, 70].

This method has been utilized to encapsulate the *L. acidophilus*La-05 strain, resulting in enhanced viability [64]. In a study by Behboudi-Jobbehdar et al. [84], the encapsulation of *L. acidophilus* using maltodextrins achieved 84% survivability. Additionally, microcapsulated *E. canintestini* S18A strain, prepared with whey protein and maltodextrin via this method, demonstrated effective viability [85].

### Extrusion

Extrusion is a low-cost, vibrational-based technique that requires minimal setup and effectively protects probiotics under various technological conditions [70]. Since the mid-1980s, extrusion has gained attention over the last two decades due to its straightforward operation, minimal disruption, and enhanced survivability [39, 57, 64]. Mechanistically, this process involves extruding a probiotic-containing hydrocolloid mixture through a nozzle into a CaCl_2_ solution, where ionic cross-linking occurs, forming gel-like microparticles that encapsulate and immobilize the probiotic cells within a protective matrix [60]. A three-dimensional droplet structure is generated when a hardening solution is employed [64]. The controlled size of the droplets can be achieved by accurately measuring viscosity, concentration, and the distance between the solution and needles. Alginate-chitosan microspheres were utilized to encapsulate *L. gasseri* and *B. bifidum* via the extrusion method, and the results demonstrated resistance against simulated gastric juice and bile solutions, indicating enhanced cellular protection during gastrointestinal transit [86]. However, these results primarily reflect cell viability rather than functionality, as the study did not assess whether the encapsulated strains retained their functional probiotic characteristics, such as adhesion, metabolite production, and antimicrobial potential, following exposure.

### Coacervation

Coacervation is a well-established and widely employed encapsulation method in the chemical, food, cosmetics, and pharmaceutical industries. This technique is characterized by its versatility, simple operating system, cost-effectiveness, and absence of organic solvents [87, 88]. It is utilized for encapsulating various volatile compounds, including probiotics. The encapsulation of *L. acidophilus*La-5 using a gelatin/arabic gum coacervation method was conducted, and the results were characterized using advanced instrumentation [87]. The findings indicated high encapsulation efficiency and significant protection for the probiotics under harsh conditions. This encapsulation strategy improved probiotic viability at different temperatures, with high viability observed at low temperatures (120 days at −18 ^∘^C) and a decrease noted at higher temperatures (45 days at room temperature) [87].

## EMs and their properties

Viability and survivability are critical factors for probiotic function. Developing an encapsulation system that ensures resistance to hostile GIT conditions while facilitating the controlled release of viable probiotics at the target site is essential [33]. Research indicates that the selection of EMs is pivotal in this regard. These materials primarily provide mechanical strength, physical shielding, and chemical stability to the cells throughout processing, storage, and digestion. They also influence probiotic viability and release kinetics, which, in turn, affect the preservation of intrinsic probiotic functionalities. Although the encapsulation matrix does not alter the intrinsic biological or metabolic properties of the probiotics, its composition and structural characteristics are vital for maintaining cell viability and ensuring that microorganisms retain their functional probiotic attributes. Selecting the appropriate material can enhance the survival and delivery of probiotics to the gut, thereby boosting their effectiveness [56, 60, 93].

**Figure 6.**
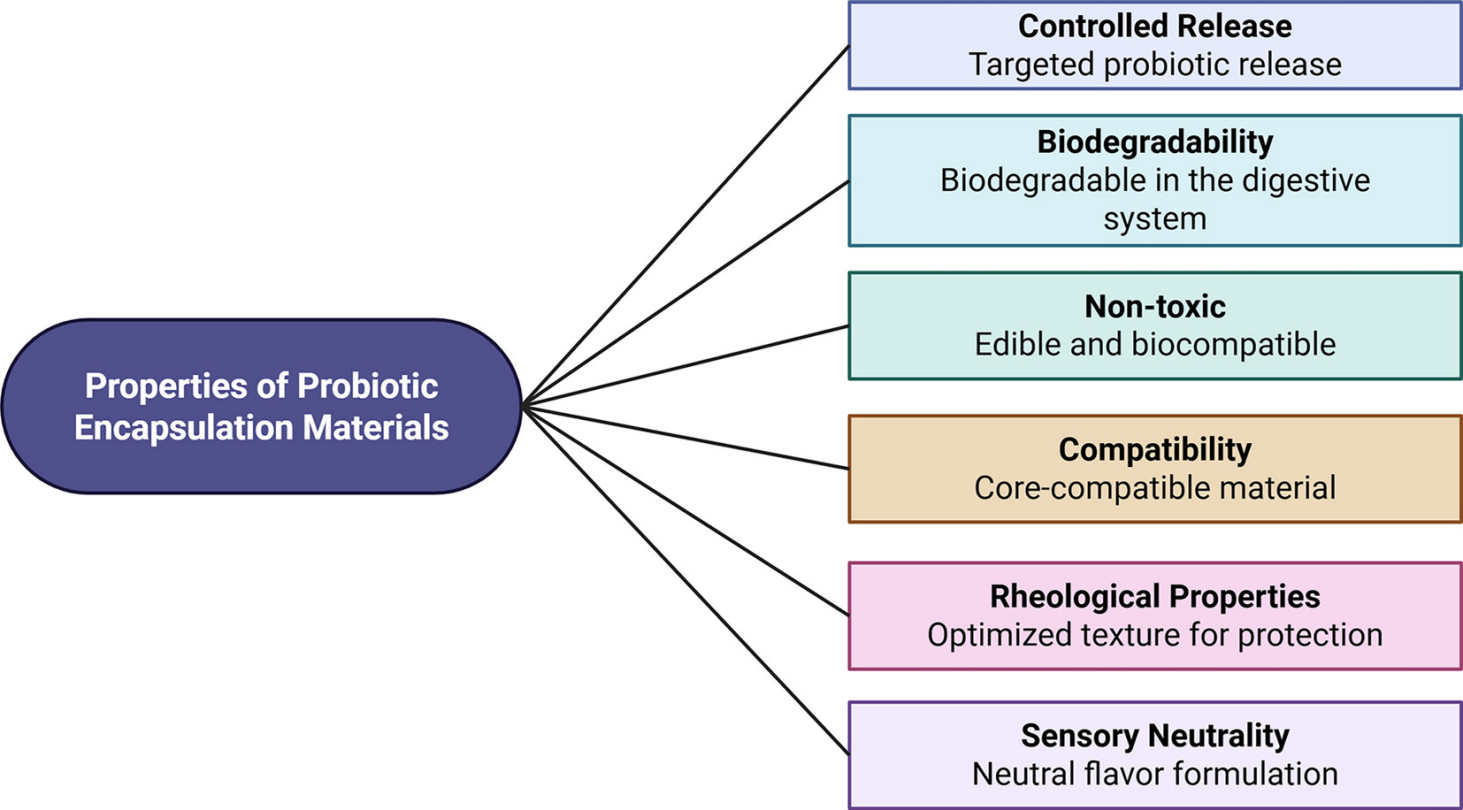
**Core performance criteria for probiotic encapsulating materials used as protective matrices or coatings.** The schematic highlights material attributes that collectively determine suitability for delivery applications, including capacity for controlled release, biodegradability under gastrointestinal conditions, non-toxicity/biocompatibility, compatibility with the probiotic core, appropriate rheological behavior for robust structure formation, and sensory neutrality to avoid altering the final product.

EMs are substances used to create a protective coating or shell around the core probiotic material. EMs consist of shells, walls, coatings, membranes, etc., and can be synthesized from both natural and synthetic substances. These materials exhibit numerous beneficial properties, including pliability, tastelessness, odorlessness, non-hygroscopicity, and film-forming potential. Furthermore, they must form a barrier without introducing any undesirable properties to the probiotics. The protection and retention of probiotic survivability during storage and transportation are also critical considerations in the selection of EMs [15, 40, 41, 93]. The choice of EMs for probiotics depends on several factors, including the specific probiotic strain, the intended application (food, pharmaceutical, etc.), and the desired properties of the encapsulated product. Compatibility between the probiotic and the EM is essential for effective functioning, protection, and targeted release in the gut under varying conditions such as enzyme presence and pH [15, 39, 59]. The general properties of probiotic EMs are summarized in Figure 6.

Before utilizing an EM in probiotic nanomaterials, certain properties must be ensured. Both natural and synthetic materials should be non-toxic, release the carrier precisely at the target site, resist adverse gut conditions, and possess sufficient residence time in the gut [33].

Materials with pH-responsive or enzymatically degradable properties are generally preferred for probiotic encapsulation. For example, polymers such as Eudragit L100 and Eudragit S100 offer acid resistance through pH-dependent solubility, ensuring targeted release in the intestine. In contrast, sodium alginate alone does not provide adequate resistance to acidic conditions; effective protection is achieved only when alginate is cross-linked with CaCl_2_ to form calcium alginate gels, which are then coated with an additional protective layer, such as chitosan or pectin. Materials like pectin and chitosan, which undergo enzymatic hydrolysis in the gut, also facilitate controlled probiotic release at the desired site [33, 60, 88]. The selection of EMs is crucial, as they define the final formulation and application of the enclosed probiotics [34]. Food-grade materials such as alginate, pectin, and starch are among the most widely used and investigated, as they effectively support the viability of probiotics [34].

Currently, combinations of different types of EMs are employed across various applications. Experimental evidence highlights the superior properties of these multi-layer EM systems. Multi-layer systems can provide enhanced protection, controlled release, and tailored properties for specific needs compared to single-layer systems [40, 59]. Multilayer EMs, whether designed as heterostructures or composite mixtures, are utilized to protect probiotics during gastrointestinal transit and ensure their targeted release in the colon, where they exert their beneficial effects. These materials, commonly composed of polysaccharides, are formulated to remain stable in the acidic gastric environment and throughout the small intestine, including the ileum. They are designed to degrade or dissolve gradually under the neutral to slightly alkaline conditions of the colon, thereby enabling controlled, site-specific release of viable probiotic cells at their primary site of action [51, 59].

### Types of EMs

**Figure 7.**
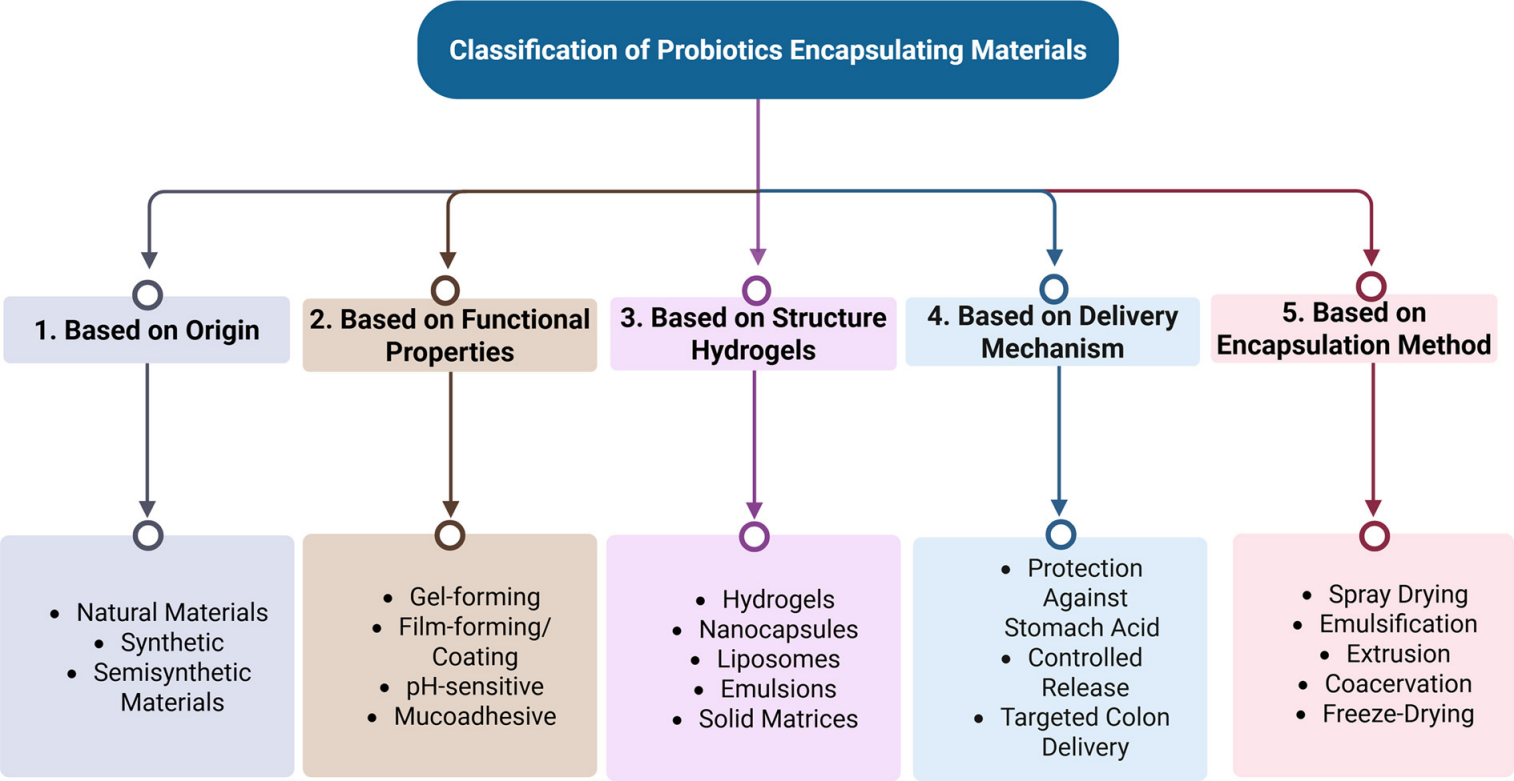
**Classification scheme for probiotic encapsulating materials and carrier architectures.** The diagram organizes materials used in delivery systems according to complementary criteria, including source (natural, synthetic, or semisynthetic), key functional properties (e.g., gel-forming, film-forming/coating, pH-sensitive, mucoadhesive), structural format (e.g., hydrogels, nanocapsules, liposomes, emulsions, solid matrices), intended delivery function (acid protection, controlled/colon-targeted release), and the processing route by which the system is produced.

Encapsulated materials are categorized based on their source, nature, processing methods, and applications. Various biological, synthetic, and natural substances are utilized to encapsulate probiotic bacteria. An overview of these materials is presented in Figure 7. Among natural sources, carbohydrates, proteins, lipids, and gum-based materials are employed due to their enhanced properties and functionalities. Key attributes of biological molecules include biocompatibility, biodegradability, enhanced properties, and large-scale availability [59, 64, 97]. The different types of EMs, along with their advantages, disadvantages, and functions, are summarized in Table 2.

### Natural materials

Natural materials are valued for their biocompatibility and biodegradability, making them essential in various applications. Most natural EMs are derived from plants, algae, and animals. The chemical composition of these natural compounds primarily consists of carbohydrates (mostly homo- or heteropolysaccharides) and proteins, while lipid-based EMs are less common. Below are widely used natural materials for probiotic encapsulation.

### Polysaccharide-based encapsulation materials

Polysaccharides represent the largest class of carbohydrates and are extensively utilized across industries. Their structure, properties, and abundance contribute to their widespread applications. Polysaccharides can be formed from various monomers, which may be identical or different.

#### Alginate

Alginate is a natural polysaccharide polymer characterized by its biocompatibility, biodegradability, and gelation properties. It contains numerous carboxylate groups that facilitate interactions with divalent ions [33, 62, 126]. In addition to its non-toxic nature, alginate can reduce immunological responses, minimize physiological disruption, and form hydrogels. Due to these remarkable properties, alginate finds applications in food, biotechnology, and biomedical sciences [35, 51, 127]. Alginate varies in type based on its constituent units and their arrangement; sodium alginate, for example, is an anionic polymer composed of β-D-mannuronic acid and α-L-guluronic acid linked by (1→4) glycosidic bonds [128].

Alginate serves as a popular encapsulating agent for probiotics due to its ability to create a stable gel matrix that protects against harsh environments and facilitates controlled release at target sites [47]. It can also be used synergistically with other materials to enhance their properties [34]. Mechanistically, sodium alginate forms an egg-box structure around probiotics, maintaining their viability and survivability during GIT transit and storage. However, the porous structure of alginate may limit its encapsulation efficiency and cause premature release of probiotics [127, 129].

Numerous studies have utilized alginate, both alone and in combination, for probiotic encapsulation, yielding promising results. Recently, Phùng et al. [130] employed sodium alginate as an encapsulating agent for three next-generation probiotic species, demonstrating enhanced properties in the presence of gastrointestinal fluids. Another study encapsulated five yeast strains within alginate beads, resulting in improved survivability for one month at 4 ^∘^C [131]. Alginate microparticles have also been used to encapsulate *Bacillus licheniformis*, aiding in the targeted release of probiotics within a simulated shrimp digestive tract and demonstrating probiotic survivability for 15 days at 4 ^∘^C [132].

**Table 2:** Summary of various encapsulating materials for probiotic delivery: Properties, advantages, limitations, and proposed applications

**Materials**	**Properties**	**Composition**	**Advantages**	**Disadvantages**	**Applications**	**References**
Alginate	Biocompatible, biodegradable, non-toxic, and water-soluble.	β-D-mannuronic acid and α-L-guluronic acid	It is pH-dependent and has a gel-like structure. The preparation process is easy and gives a stable matrix.	It is porous and requires another material for safe encapsulation. It is also affected by low pH.	It is widely used in the food, biotechnology, and biomedical industries. It is also employed as an encapsulating agent.	[33, 62, 98]
Chitosan	A natural, non-toxic, biocompatible, biodegradable polymer with mucoadhesive potential.	Glucosamine and N-acetylglucosamine	It is a pH-dependent polymer and can release the substance at the target sites.	It can easily be degraded by low pH, and sometimes swelling occurs.	It has potential usage in the biotechnology, medicine, food, and agriculture sectors.	[33, 60, 99]
Pectin	A non-toxic, biocompatible polymer possesses significant potential for emulsification, thickening, and stabilization.	Galacturonic acid	It can be modified to enhance the attachments and survivability of probiotics.	The greater hydrophobicity of pectin can cause swelling and release of probiotics.	It has applications in biomedical, drug delivery, wound healing, and food industries.	[33, 100, 101]
Gums	Abundant, biocompatible, and viscous.	D-glucose, D-glucuronic acid, and L-rhamnose	It is a non-toxic, stable, and cheap polymer.	It has lower mechanical strength and a high gelling temperature.	It is used in targeted delivery, food, and biomedical industries.	[59, 102, 103]
Cellulose	It has high tensile strength, is biodegradable, and is a water-insoluble polymer.	Linear polymer of glucose units	It is a non-toxic, easily available, and environmentally friendly substance.	It is not digested by humans.	It is used in the textile, food, and biomedical industries.	[39, 104, 105]
Starch	It is a viscous, biocompatible, and biodegradable polymer.	Glucose units	Affordable and readily available, with strong encapsulation potential.	Porosity and high hydrophilicity.	It is used in food, biotechnology, and agriculture.	[106, 107]
Agarose	Natural, biocompatible, and biodegradable (non-biodegradable in the animal body), and gelling potential.	D-galactose and anhydro-L–galactose	The easy availability, affordability, and enhanced oxygen-carrying capacity of this material contribute to its significance in various applications.	Temperature-dependent, with high gelation potential.	It is used in food, agriculture, and biotechnological applications.	[59, 108]
Pullulan	It is a natural, non-toxic, biocompatible, and linear polysaccharide.	Maltotriose units	Tasteless, adhesive, and biodegradable properties.	High OH group content allows easy modification.	Pharmaceutical, biomedical, and food industries.	[109, 110]
Dextran	Biocompatible and biodegradable polysaccharide.	D-glucopyranose	It forms a gel-like structure and has many OH groups.	Dextranase enzymes can degrade it, causing early release.	Biomedical, drug delivery, and food industries.	[111, 112]
Gelatin	Protein-based natural polymer has thickening, gelation, and hydrophilic properties.	Glycine, proline, and hydroxyproline	It is hydrophilic, biocompatible, and cheaply available.	It has low water resistance and poor mechanical properties.	It is widely used in the biomedical, pharmaceutical, cosmetics, and biotechnology industries.	[47, 113, 114]
Casein	It has the properties of gelation, emulsification, and foaming.	Proteins and other substances in traces	Easily available, cheap, biocompatible, and biodegradable.	It is highly sensitive and lacks durability.	Its area of usage includes the food, beverage, and biotechnology sectors.	[115, 116]
Zein	It is a stable, biocompatible, and biodegradable protein.	Non-polar amino acids, i.e. glutamic acid, leucine, proline, and alanine	It is abundant, cheap, and has sustained release potential.	It has lower water solubility and greater potential for aggregation.	It is used in drug delivery, food, and biotechnology applications.	[117, 118]
Eudragit	Its pH-dependent biocompatible, and non-biodegradable.	It is a synthetic polymer of methacrylate and methacrylic acid	It has controlled releases and gives smooth surface properties.	The pH-dependent nature may cause bursts and early release.	Extensively used in biomedical, drug delivery, and pharmaceutical areas.	[33, 119, 120]
Carrageenan	It is a natural, biocompatible, and stabilizing polymer.	β-D-galactose and α-D-galactose	It acts as a protective barrier and depends on temperature.	It interacts with other substances and can cause bloating.	It is used in food, cosmetics, drug delivery, and biomedical areas.	[121, 122]
Carboxymethyl cellulose (CMC)	It is a non-toxic, biocompatible, abundant, and synthetic polymer.	Carboxymethyl groups	It is cheap and has lipid and oxygen barrier properties.	It has a weak water barrier potential.	It has a role in agriculture, drug delivery, and biomedical sectors.	[123, 124]
Polyvinyl alcohol (PVA)	It is a non-toxic, biocompatible, and synthetic polymer.	Vinyl alcohol units	It has stability, resistance, and flexibility benefits.	It has a sensitivity to moisture and heat.	It is used in the paper, textile, food, and agriculture sectors.	[73, 125]

#### Chitosan

Chitosan is a natural linear biopolymer composed of glucosamine and N-acetylglucosamine units linked by β-(1→4) glycosidic bonds. It is characterized by its non-toxic, biocompatible, and biodegradable properties, with the number of constituent units varying according to the source and preparation method [133]. Chitosan has numerous applications in tissue engineering, drug delivery, and the food industry, serving as an encapsulating agent for bioactive compounds and probiotics [15, 42, 47]. Structurally, the positively charged moieties of chitosan electrostatically bind to other polymers, entrapping probiotics and providing protection under harsh conditions [33, 70]. However, its pH-dependent nature can adversely affect the viability of encapsulated materials in the acidic environment of the GIT [55, 134].

The addition of outer core materials can enhance the survivability of chitosan-encapsulated probiotics in acidic conditions. However, the use of crosslinkers in multi-layer protection may negatively impact probiotic viability, potentially due to swelling formation [33, 135]. Chitosan-encapsulated probiotics can achieve targeted release in the intestine (alkaline pH), where higher pH levels cause the chitosan layers to swell and degrade, subsequently releasing the encapsulated probiotics. Notably, the positively charged moieties of chitosan interact with the negatively charged sialic acid moieties of mucin, enhancing probiotic attachment in the intestine [15, 42, 136]. Chitosan-encapsulated probiotics demonstrate greater release in the colon, facilitated by colonic pH, which causes the degradation of the chitosan matrix and increases the number of probiotics in the colon [33, 39, 137].

Currently, many probiotic strains are encapsulated with chitosan, yielding promising results regarding viability and survivability. For example, the encapsulation of *L. casei* ATCC 39392 and *B. bifidum* ATCC 29521 using a chitosan-alginate-inulin mixture resulted in enhanced survivability under simulated gastric conditions [138]. Similarly, chitosan- and dextran-based hydrogels encapsulated L. *acidophilus*, demonstrating superior survivability compared to non-encapsulated cells [112]. Peñalva et al. [139] encapsulated *L. plantarum* (CECT 220 and WCFS1 strains) using casein-chitosan microparticles, achieving higher loading capacity and improved probiotic survivability under harsh gastric conditions.

#### Pectin

Pectin is a plant-derived heteropolysaccharide composed of 17 different monomers linked by α-1,4 bonds. It is classified into high- and low-methoxy pectin based on the presence of methyl groups [100, 101, 140]. As a natural polymer, pectin exhibits properties of biocompatibility, stability, and stress resistance, making it suitable for various applications in the food and biotechnology industries. Pectin’s ability to bind with mucin also contributes to its targeted release capabilities [33]. During encapsulation, pectin protects probiotics from harsh environmental conditions and enhances their viability during storage. However, lower mechanical strength and larger gaps in the capsule may limit its applications [141]. During probiotic encapsulation, pectin provides protection and enhances survivability within the GIT and during storage [142]. Various substances, including gut microbial enzymes, influence the release of probiotics [143]. Several studies have demonstrated the encapsulating potential of pectin with promising results. For instance, Li et al. [144] used pectin to encapsulate *B. breve*, achieving over 99% efficiency. The combination of pectin and zein nanoparticles (ZNPs) as encapsulating agents for probiotics, such as *L. plantarum* 550, resulted in greater survivability (>95%) under harsh conditions. Pectin as the outer layer enhanced heat stability (0.61 log CFU/mL loss), while ZNP improved storage stability (0.21 log CFU/mL loss) [91]. Recently, pectin in combination with other encapsulating agents was utilized to protect *L. plantarum* WCFS1, revealing high survival rates for probiotics under challenging conditions [141].

#### Starch

Starch is a natural polymer composed of glucose units linked together by glycosidic bonds. Depending on the number of glucose units and the branching positions, starch can exist in two forms: amylose, which is linear and water-soluble, and amylopectin, which is extensively branched and water-insoluble. These forms typically constitute 20%–30% and 70%–80% of starch, respectively [42, 145]. Starch serves as the primary storage form of energy in plants and possesses diverse properties that render it crucial for various applications. The presence of hydroxyl (OH) groups on starch molecules facilitates their modification into multiple forms. Among its various applications, starch is also utilized as an encapsulating agent [47, 107]. Materials encapsulated with starch exhibit improved resilience against harsh conditions, including those encountered in the GIT and during processing. However, the porous structure of starch can lead to the premature release of encapsulated substances, thereby limiting its applications [59, 64, 146].

Extensive research has been conducted on starch as an EM for probiotics, demonstrating effective protective and delivery properties across various studies. Multiple formulations derived from starch or its derivatives are employed as probiotic encapsulating agents. These coatings provide essential protection, preserve cell viability, and help maintain stable viable counts during processing and storage [56, 147]. The survivability of encapsulated probiotics can be enhanced when starch is combined with other materials [39, 42]. Noman et al. [147] investigated the effects of starch-based nanoparticles on the viability and stability of probiotics under adverse conditions and found that encapsulated probiotics exhibited higher viability compared to their non-encapsulated counterparts. Similarly, Khosravi Zanjani et al. [138] utilized alginate-gelatinized starch microcapsules coated with chitosan for the encapsulation of *Lactobacillus casei* and *Bifidobacterium bifidum*, reporting enhanced protection in the presence of simulated intestinal juice.

#### Dextran

Dextran is a homopolysaccharide composed of repeating units of α-1,6 D-glucopyranose, produced by specific LAB. While the primary linkage is α-1,6, other linkages such as α-1,3 and α-1,4 are also present, all connected by glycosidic bonds. The type and extent of branching vary depending on the bacteria involved in its production [111, 148]. The incorporation of various cross-linkers allows dextran to form gel-like structures. Its multiple hydroxyl groups contribute to its significance in biomedical applications [59]. Due to its probiotic encapsulation potential, dextran is widely employed because of its biocompatibility and hydrophilic properties. The dextranase enzyme degrades dextran, which can influence the release of encapsulated bacteria depending on dextranase availability. This characteristic allows dextran hydrogels to be utilized as vehicles for colon-specific drug delivery [59]. The use of chitosan and dextran sulfate hydrogels with genipin crosslinker was found to reduce the viability of *L. acidophilus* by 3.6 log CFU/mL, attributed to the cross-linking conditions or specific interactions between genipin and the polymers, which formed a dense structure that caused viable cell reduction, rather than any inherent toxicity of dextran sulfate, which is generally regarded as a safe EM [112].

#### Agarose

Agarose is a linear polysaccharide composed of β-1,3-linked D-galactose and α-1,4-linked 3,6-anhydro-L-galactose units, known for its gel-forming potential [59]. The formation of agarose gel is influenced by factors such as temperature and oxygen levels; at low temperatures, a hydrogel forms, while higher oxygen carrier capacity makes it suitable for microencapsulation [39, 59]. Agarose exhibits temperature-dependent gelling behavior, forming a hydrogel at approximately 40 ^∘^C, which is advantageous for encapsulation applications. To achieve a higher gelation temperature or modified gel properties, agarose can be blended with other polymers to adjust its thermal and mechanical characteristics [108]. Its gel network provides a stable physical matrix that protects cells from environmental and gastrointestinal stresses. However, agarose’s non-degradable nature under gastrointestinal conditions limits its use as an encapsulating agent. Moreover, studies comparing its efficacy to other biodegradable substances regarding release behavior, colon-targeted delivery, and biocompatibility can enhance its potential. It is important to note that probiotic viability alone is not a sufficient indicator for a promising encapsulating agent, necessitating further studies [59, 108, 113]. Despite this, agarose’s properties, including gelling potential, high hysteresis, gel reversibility, and odorless taste, make it a significant agent in the food and pharmaceutical industries [39].

Agarose is also utilized as a probiotic encapsulating agent, offering protection during processing and harsh gastrointestinal conditions. Studies have shown that agarose-encapsulated probiotics maintain high survivability and stability. A novel approach employing chitosan-coated agar-gelatin gel particles was applied to encapsulate *Lactobacillus plantarum* NCIMB 8826, demonstrating effective protection during exposure to gastric juice (pH 2.0) over 2 h [149]. Similarly, *B. pseudocatenulatum* CECT 7765 was encapsulated with agarose and exhibited greater survivability under simulated *in vitro* digestion conditions [59]. Although agarose is favored as an encapsulating agent due to its mechanical strength and stability, its non-degradable nature (as agarose-degrading enzymes are absent in the animal body) can limit its potential [59].

#### Cellulose

Cellulose is a linear biopolymer composed of β-D-glucose units connected by β-(1–4) glycosidic bonds. Its high number of hydroxyl groups contributes to its polar nature, enhancing its range of applications [39, 42, 59]. Cellulose is an amphiphilic, non-toxic, renewable, biocompatible, biodegradable, semi-crystalline, and environmentally friendly material, characterized by excellent mechanical strength and remarkable surface properties [42, 47, 104]. It finds extensive use in various industries, including healthcare, food, electronics, and printing. In healthcare, cellulose is utilized in tissue engineering and drug delivery systems, while in the food industry, it serves as a stabilizing, thickening, and bulking agent. Its environmentally friendly nature makes it an integral component of printing systems [105]. Cellulose-based polymers are widely used in microencapsulation systems. In this context, cellulose nanofiber (CNF) and cellulose nanocrystal (CNC), which are non-toxic nanomaterials, have been experimentally validated for probiotic encapsulation [42]. Due to its indigestibility, cellulose is considered a promising option for targeted drug delivery systems [104]. Recent research summarizing the various types of cellulose and their applications in probiotics has been conducted by Yang et al. [104].

Sabio et al. [150] developed a cellulose-based biomaterial for encapsulating probiotics (*Lactobacillus fermentum* or *Lactobacillus gasseri*) and found that the encapsulated cells remained active and viable, demonstrating potential for treating skin infections and wounds. A CNF and inulin-incorporated carboxymethyl cellulose (CMC)-based probiotic nanocomposite developed by Zabihollahi et al. [97] achieved a 36% increase in the viability of encapsulated cells. Conversely, Salimiraad et al. [113] reported a negative correlation between the survival of selected probiotics and storage time after encapsulating *L. casei* (survival rate of 6.01 log CFU/g) and *B. coagulans* (survival rate of 6.35 log CFU/g) in cellulose-nano chitosan-gelatin films. Nevertheless, these final counts remain within the acceptable probiotic range, indicating that poor encapsulation performance is not a valid conclusion.

#### Pullulan

Pullulan is a linear, water-soluble, neutral polysaccharide composed of maltotriose units linked by α–1,6 bonds. It is characterized by its non-toxic, non-carcinogenic, biodegradable, adhesive, and film-forming properties, along with a white appearance devoid of odor and taste [110, 151]. The abundance of hydroxyl groups in pullulan’s structure allows for significant modification potential, making it suitable as a probiotic encapsulating and delivery agent [59, 152].

Inherent properties and the potential to form nanocomposites render pullulan highly valuable. When combined with other materials during probiotic encapsulation, it can enhance the survivability of enclosed cells under harsh conditions [109, 152, 153]. Studies have demonstrated pullulan’s protective role for probiotics against oxidative stress and moisture, thereby improving their shelf life and stability [109, 151, 154]. A multi-layer encapsulation system comprising pullulan nanofibers and two electrospun poly-lactic-co-glycolic acid (PLGA) layers for the probiotic *Lactobacillus rhamnosus* GG (LGG) showed enhanced viability and prolonged storage potential [153]. In another study by Ma et al. [74], a blend of gum Arabic and pullulan nanofibers prepared via electrospinning was employed as an encapsulating agent for *Lactobacillus*, resulting in greater survivability (85.38%–97.83%) compared to freeze-drying (80.92%–89.84%) and viability during 4 ^∘^C storage for 28 days. Pullulan, classified as GRAS, possesses anti-bacterial, anti-carcinogenic, and anti-viral properties, making it an essential agent in the food, cosmetics, biomedical, and pharmaceutical sectors [152].

#### Gums

Gums are complex polysaccharides characterized by diverse structures and properties. Gellan gum, a linear exopolysaccharide, consists of repeating tetrasaccharide units (1,3-β-D-glucose, 1,4-β-D-glucuronic acid, 1,4-β-D-glucose, and 1,4-α-L-rhamnose). Its attributes include gelation, emulsification, thickening, and stabilization, making it valuable in the food, pharmaceutical, cosmetic, and biotechnological industries [102, 155]. Additionally, gums are non-toxic, stable, less viscous, and possess retention properties [59]. Based on their sources, gums can be categorized as plant-based, exudate, and microbial gums, which are extracted from plants (such as seeds and bark), secreted by plants in response to stress or injury, and obtained from microorganisms, respectively [103, 156].

The intrinsic properties of gums render them suitable for encapsulating various substances, including probiotics. Xanthan gum (XG) is an anionic biopolymer known for its excellent biocompatibility and gelling properties, as well as its stability against heat and acids [64]. For probiotic encapsulation, gum arabic is typically used in conjunction with other polymers. Gums form a protective coating around probiotics, safeguarding them during the harsh conditions of the GIT and industrial processes [93]. They also enhance the stability and survivability of probiotics during storage and utilization in the food industry [102]. While gums offer advantages such as gelation, biocompatibility, and accessibility, they also present challenges, including poor mechanical strength, lower stability, and high gelling temperatures [59]. Saeed et al. [157] demonstrated that a combination of alginate-carrageenan gums effectively encapsulated *L. acidophilus* and *L. casei*, resulting in increased probiotic viability in cottage cheese stored at 4 ^∘^C for 28 days. Similarly, Pandey et al. [158] utilized xanthan and guar gum as encapsulating agents for *L. plantarum* 299v, achieving enhanced viability during storage and controlled release.

### Protein-based EMs

Various biological and protein-based EMs are employed to protect probiotics. Proteins derived from both plant and animal sources are utilized, although animal-based proteins may exhibit allergenic properties. Protein denaturation can enhance the mechanical properties of probiotics, while the aggregation of denatured proteins increases their strength and elasticity due to disulfide bonds [8, 59]. Probiotics coated in denatured proteins can improve their effectiveness under gastric conditions. Protein-based materials used in microencapsulation are categorized into animal and plant-derived proteins.

### Animal-based proteins

Animal-derived proteins, despite their potential allergenic properties, are employed as encapsulating agents due to their beneficial characteristics. Key animal-based proteins are discussed below.

#### Gelatin

Gelatin is a water-soluble, degradable biopolymer with a complex structure composed of polypeptide chains. It exhibits amphoteric and thermoreversible gelling properties, making it suitable for encapsulation either alone or in combination with other materials [47, 64]. Gelatin predominantly consists of glycine, proline, and hydroxyproline, which together constitute approximately 57% of its structure. Additionally, gelatin possesses cationic and ionic properties, allowing it to be combined with other substances [15]. There are two commercially available types of gelatin, Type-A and Type-B, which respond differently to pH levels. Gelatin’s cost-effectiveness contributes to its significant applications in medicine and food industries, including food packaging, emulsification, and encapsulation [159].

The recent interest in multilayer encapsulation is due to its enhanced resistance and release properties in the presence of gastric and intestinal juices [15, 64]. Gelatin’s gelation potential, biodegradable nature, and biocompatibility further support its role as an encapsulating agent. Its film-forming ability and biocompatibility contribute to its protective capabilities[160]. However, certain limitations are associated with gelatin, including high solubility, pork origin (which is not permissible for Muslims), and limited thermal stability [59, 114].

Research evaluating the use of gelatin in combination with sodium alginate for encapsulating *L. acidophilus* LA-5 probiotics during baking and storage conditions indicated higher survivability of encapsulated bacteria over seven days compared to free cells, suggesting its potential as a baking enhancer [161]. The encapsulation of *Kluyveromyces lactis* using gelatin hydrogels demonstrated improved survival and physicochemical properties [162]. Rama et al. [163] employed ricotta whey and gelatin as encapsulating agents for *L. acidophilus* LA-5 and *B. lactis* BB-12 using a spray-drying method, revealing less reduction in viability compared to non-encapsulated cells.

#### Casein

Casein, a milk protein comprising 94% proteins and 6% low molecular weight compounds, exhibits gelation, foaming, and emulsification properties. It is utilized in various applications, including food, beverages, biotechnology, tissue engineering, drug delivery, and biomedical fields [115, 164]. Casein’s ability to form micelles allows it to entrap and protect various substances, making it a suitable agent for probiotic encapsulation [116, 164, 165]. In addition to providing protection during gastrointestinal transit, casein facilitates targeted release in the intestine [78, 165, 166]. Casein-based encapsulation systems maintain probiotic viability during gastrointestinal transit and storage by forming stable matrices that shield cells from acidic and enzymatic degradation. Moreover, casein can be combined with other biopolymers, such as alginate or chitosan, to enhance mechanical strength and modulate release kinetics, thereby establishing itself as a versatile candidate for probiotic delivery applications [164, 165].

In contrast to polysaccharide-based materials, such as alginate or chitosan, which primarily provide pH-dependent protection, casein offers a protein-based matrix capable of forming micelles that entrap and stabilize probiotic cells. This structure allows for the gradual release of probiotics as the matrix undergoes enzymatic degradation. Specifically, casein is digested by gastrointestinal proteases, such as pepsin in the stomach and trypsin in the intestine, leading to controlled disintegration and site-specific release of viable probiotics. Comparative studies have demonstrated that casein-based systems can achieve equal or greater cell viability during storage and simulated digestion compared to conventional polysaccharide matrices. Consequently, casein not only provides physical protection but also facilitates targeted delivery through enzyme-triggered release mechanisms [115, 165]. Peñalva et al. [139] employed casein-chitosan microparticles to encapsulate *L. plantarum* (CECT 220 and WCFS1) in an *in vivo* model, revealing higher survivability and safe release in the distal ileum. Similarly, encapsulating *Lactobacillus* F19 and *_B.* Bb12 with casein through an enzymatic gelation process resulted in greater survivability during three months of storage under optimized conditions [59].

#### Whey proteins

Whey proteins are globular, water-soluble proteins consisting primarily of β-lactoglobulin, α-lactoalbumin, and immunoglobulins, which are derived from whey during cheese production [89]. Their varied structures provide desirable properties such as high digestibility, gelation, antioxidant activity, and antihypertensive effects, making them essential in the food, biotechnology, and pharmaceutical industries [89, 167]. They serve as effective encapsulating agents for bioactive compounds and probiotics, enhancing the shelf life of encapsulated materials and acting as delivery vehicles in pharmaceutical applications [78, 168]. The ability of whey proteins to gel at low temperatures without cross-linkers is crucial for probiotic encapsulation under industrial conditions [59]. For instance, encapsulation of L. *fermentum* 39-183 with whey protein isolates demonstrated 86% survivability in *ex vivo* conditions (pH 2.0 for 3 h), compared to a complete loss of viability in free cells [169]. The combination of whey proteins with other encapsulating agents further enhances protection; for example, the use of whey proteins with calcium alginate provides superior protection for probiotics [170]. Additionally, a whey protein isolate-lignin complex was utilized as an encapsulating agent for *L. reuteri* KUB-AC5, yielding higher protection and survival rates for the encapsulated probiotics *in vitro* [171].

### Plant-based proteins material

Plant-based proteins are increasingly recognized as EMs for probiotics, offering a sustainable approach for applications in the food, pharmaceutical, and cosmetic industries [59].

#### Zein

Zein is a biodegradable, thermostable, hydrophobic protein derived from gluten and is widely utilized across various industries [59]. Recognized as GRAS, zein’s ease of modification enhances its applicability in food, pharmaceuticals, biotechnology, and drug delivery processes [117, 172]. Its versatility in encapsulation technology significantly enhances probiotic viability in the presence of gastric juice [59]. For example, *B. subtilis* was successfully encapsulated with zein and soluble soybean polysaccharide (SSPS), demonstrating increased viability during GIT, storage, and pasteurization. Results indicated a 3.13-fold increase in simulated gastrointestinal digestion, a 3.20-fold increase during pasteurization, and a 1.50-fold increase during storage conditions [118].

#### Pea proteins

Pea protein isolates possess several beneficial properties, including low allergenic potential, sustainability, emulsifying capabilities, and film-forming capacity, making them valuable encapsulating agents for various food and biotechnology applications [88, 173]. Research has explored the encapsulation of probiotics with pea proteins. For instance, encapsulation of *L. reuteri* ATCC 53608 with pea proteins and alginate resulted in enhanced survivability under gastrointestinal conditions in studied mice [59]. A recent study by Saiz-Gonzalo et al. (2025) demonstrated the microencapsulation of *Lacticaseibacillus rhamnosus* GG (ATCC 53103) with pea protein, showing significantly greater survivability compared to non-encapsulated cells, where 50% of cells were lost under gastric conditions. In contrast, pea-encapsulated cells exhibited enhanced viability at higher pH levels (5–7) [212].

#### Soy proteins

Soy proteins and their isolates exhibit properties that render them essential across diverse industries. Their solubility, stabilization potential, gelation, and film-forming capabilities are notable attributes. Consequently, soy proteins are employed as encapsulating agents for bioactive compounds, fish oil, and probiotics [88, 174]. Soy protein microcapsules have demonstrated improved viability for encapsulated probiotics in gastric conditions [59]. A recent study by Babot et al. [174] utilized soy protein isolate-alginate microcapsules to encapsulate *Ligilactobacillus salivarius* CRL2217, yielding promising results regarding the protective potential of the EM, which enhanced probiotic survivability in gastric juice (low pH) and protected against proteolytic activity.

### Synthetic EMs

Despite the extensive use of natural substances in encapsulation, they often present limitations such as tedious extraction processes, limited solubility, and complex processing protocols. This has led to the development of synthetic polymers as EMs. Synthetic materials offer enhanced protection for encapsulated agents, such as probiotics, in harsh conditions. The primary advantages of synthetic polymers include controlled release, higher stability, and reduced reactivity.

#### Eudragit

Eudragit is a synthetic polymer composed of methacrylic acid and methacrylate monomers in varying proportions. This polymer exhibits pH-responsive characteristics and has applications in food, agriculture, biomedical, and pharmaceutical industries [8, 33, 119]. Its controlled release potential has been utilized in the coating of tablets and bioactive compounds. Notably, the two common pH-responsive polymers, L100 and S100, exhibit resistance under gastric conditions [119, 126]. As a probiotic encapsulating agent, Eudragit provides protection during storage and facilitates targeted delivery, with these properties further enhanced when combined with additional EMs [33, 42, 175]. However, despite its advantages, challenges such as food interactions, processing difficulties, and thermal instability may limit its applications [63, 64, 119].

Eudragit is frequently employed in probiotic encapsulation. For instance, Ansari et al. [176] utilized calcium alginate-chitosan and Eudragit S100 nanoparticles as EMs for L. *rhamnosus*, demonstrating enhanced survivability under simulated gastric and intestinal juices (pH 1.5 and 7.5). Similarly, *L. rhamnosus* GG was encapsulated in Eudragit^®^ S100 microparticles for effective colonic release, and this approach showed promising results, with greater viability in the gastrointestinal environment after 6 h of incubation [175]. Furthermore, the encapsulation of *L. casei* and *L. bulgaricus* using chitosan and Eudragit S100 resulted in a decrease in viability from 6.0 × 10^6^ and 7.2 × 10^6^ (on the first day) to 4.1 × 10^5^ and 5.3 × 10^6^ (on day 32) under gastrointestinal conditions [177].

#### Carrageenan

Carrageenan is a high molecular weight linear polysaccharide composed of alternating β-D-galactose and α-D-galactose units linked by glycosidic bonds [121, 178]. Its notable properties—gelation, viscosity, and thickening ability—enhance its applications across biomedical, food, cosmetic, and biotechnological sectors [122]. Based on structural arrangement and repeating unit patterns, carrageenan is categorized into lambda (λ), kappa (κ), or iota (ι) forms [47, 64, 179]. Temperature significantly influences its structure and functionality, making it a vital biomaterial for delivery systems across varying thermal conditions [64].

Probiotics encapsulated in carrageenan exhibit improved stability, survivability, and viability during storage and simulated conditions [64, 66]. Recently, Saeed et al. [157] encapsulated *L. acidophilus* and *L. casei* using alginate-carrageenan gums, resulting in higher viability in cottage cheese. Similarly, Sogut et al. [180] demonstrated that encapsulating mixed cultures of *Lactobacillus* spp. and *Bifidobacterium* spp. with carrageenan resulted in enhanced survivability and cell counts. Hydrogels made from sodium alginate and carrageenan were employed for encapsulating *L. acidophilus* ATCC-4356, achieving a higher survival potential (3 log reductions) compared to free cells (6 log reduction) [66]. Overall, research on carrageenan encapsulation primarily focuses on evaluating physical protection and cell survivability under gastrointestinal conditions, while limited information exists regarding the behavior of cells post-release from EMs.

#### PVA

PVA is a water-soluble, biocompatible, and non-toxic synthetic polymer that can be easily modified for various applications, including encapsulation, drug delivery, and tissue engineering. PVA is characterized by its odorless and tasteless nature, chemical resistance, and stability [125, 181]. In addition to its diverse applications, PVA serves as a probiotic encapsulating agent. A study utilized a combination of PVA and PVA/SF nanofibers to encapsulate L. *plantarum*, resulting in greater survivability for 2 h under gastric conditions [73]. In another study, a gelatin–PVA hydrogel successfully trapped *Lactiplantibacillus plantarum* spp. CM-CNRG TB98, achieving a probiotic loading capacity and a survival rate exceeding 94% in the intestine [182]. The *Escherichia coli* strain Nissle 1917 (EcN) was encapsulated in composite mats of cellulose acetate and PVA, demonstrating improved survivability of probiotics under simulated digestive conditions [183]. The addition of chitosan to PVA enhanced probiotic viability; *Bifidobacterium animalis* subsp. *lactis* Bb12 encapsulated in chitosan/PVA, along with inulin as a prebiotic, exhibited greater survivability in simulated gastric and intestinal juices [184]. However, current research on PVA encapsulation predominantly addresses probiotic protection during gastrointestinal conditions, with limited knowledge of cell behavior post-release, highlighting the need for comprehensive assessments of post-release functionality to ensure that encapsulated probiotics retain their biological efficacy.

#### CMC

CMC is a widely utilized packaging material known for its non-toxic, biodegradable properties, as well as its ability to form films and serve as an efficient barrier to oxygen and lipids [97, 104]. While the abundance of hydroxyl groups contributes to its functionality, it also results in poor water barrier properties, limiting its applications [97]. Incorporating CMC into nanocomposites can mitigate these limitations by forming barriers that restrict water ingress [185, 186]. The flexibility and biocompatibility of CMC are crucial for its applications in agriculture, food packaging, drug delivery, and encapsulation, providing protection during storage, delivery, and processing [104, 124].

The probiotic *L. plantarum* was encapsulated using CNF and inulin-incorporated CMC nanocomposites, enhancing protection and antibacterial potential [97]. An improved survivability of the encapsulated probiotic (*L. acidophilus*) was observed under simulated GIT conditions using a layer-by-layer approach with polyelectrolytes, chitosan, and CMC, achieving a survivability of 6 log CFU/500 mg compared to near-complete mortality of non-encapsulated cells [62]. Additionally, a layer-by-layer approach combining sodium alginate, whey protein isolate fibrils, and CMC for encapsulating *L. plantarum* 90 demonstrated a survivability of 6.65 log CFU/mL after 48 h of fermentation [92].

## Applications of encapsulated probiotics

**Figure 8.**
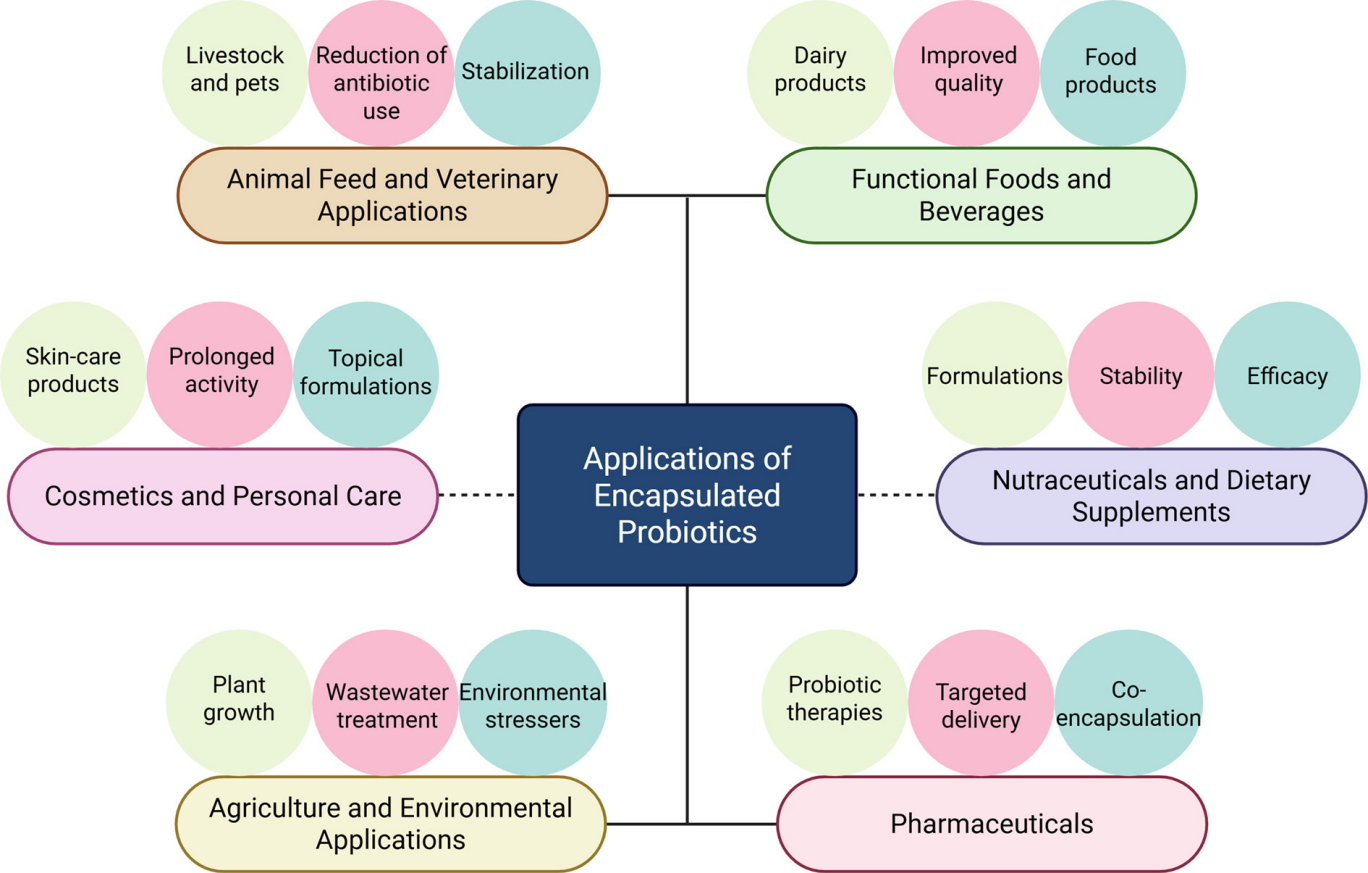
**Cross-sector application landscape for encapsulated probiotics.** The schematic summarizes major end-use domains—functional foods and beverages, nutraceuticals/dietary supplements, pharmaceuticals, animal feed/veterinary use, cosmetics/personal care, and agriculture/environmental applications—illustrating how encapsulation supports sector-specific goals such as stability, efficacy, improved product quality, targeted delivery, prolonged activity, and performance under environmental stressors.

Encapsulation enhances the protection and viability of encapsulated materials, broadening their applications. The effectiveness of encapsulated probiotics varies depending on the nature, type, and method of encapsulation. Encapsulated probiotics find extensive applications across food, agriculture, biomedical, drug delivery, health, and biotechnological industries. A summary of the diverse applications of encapsulated probiotics is presented in Figure 8.

The intrinsic properties of EMs—such as pH sensitivity, thermal stability, and controlled-release behavior—significantly enhance probiotic viability and survivability, facilitating targeted delivery within the GIT. A summary of the probiotic encapsulation methods, materials used, duration, and outcomes is provided in Table 3. Key areas of probiotic encapsulation are discussed below.

**Table 3:** Summary of various probiotics encapsulated with different materials: Methods, outcomes, and potential applications

**Probiotics**	**Methods**	**Materials**	**Outcomes**	**Conditions**	**Applications**	**References**
*L. acidophilus* LA-5	Emulsification	Inulin, hi-maize and rice bran	Enhanced efficiency and greater viability.	At 7 ^∘^C, viable for 120 days.	To improve the storage time	[82]
*L. plantarum* ATCC:13643	Extrusion	CMC/k-carrageenan	The survival rate was 7.30 log CFU/g.	2 h in simulated gastric fluid	Probiotic delivery to the colon	[201]
*B. longum* BIOMA 5920	Electrostatic droplet	Alginate and human-like collagen (HLC)	Higher viability and 90%–92.2% efficiency.	Simulated gastric fluid and 3 weeks of storage at 4 ^∘^C.	Novel delivery system for oral administration of bioactive compounds	[202]
*L. rhamnosus*	Freeze-drying	Alginate	Mechanical support and protection. Higher survivability.	6 weeks at 25 and 4 ^∘^C	Nutraceutical microcapsule	[35]
*L. plantarum* LAB12	Extrusion	Alginate, xanthan, and gums	78.34%–81.63% efficiency	(>7 log CFU/g) at pH 6.8	Functional food ingredient with health claims	[203]
*Bacillus coagulans*	Microwave drying	Bacterial nanocellulose and pectin	Improve probiotics protection and resistance to harsh conditions. 1.3–1.8 log CFU/g loss.	4 ^∘^C, and −20 ^∘^C	Oral probiotics under various temperatures and storage	[204]
*L. plantarum* MB001	Hydrogels and emulsions	Alginate with an ionic cross-linker	High survival rate and controlled release. It offers long storage and improves biostability.	4 ^∘^C, 25 ^∘^C, −20 ^∘^C	Foods and drug supplement	[205]
*L. plantarum*	layer-by-layer	Zein nanoparticles and pectin	95% survival	60 days at 4 ^∘^C	Protection of probiotics in the food matrix	[91]
*L. acidophilus*	layer-by-layer/freeze-drying	Chitosan and carboxymethyl cellulose	Protection from the harsh gut conditions and greater survivability.	120 min	Simulated gastric fluid for 2 h	[62]
*L. rhamnosus* GG	Spray-drying	Whey protein and resistant starch matrices	Enhance survival and have high viability.	5 weeks at 4 ^∘^C and 25 ^∘^C	Enhanced stability and shelf life	[196]
*Lactobacillus casei* Shirota, *L. plantarum* Lp33, and Lp17	Emulsion	Co-encapsulation of alginate combined with potato starch, plantago psyllium, and inulin	Give gastrointestinal protection. 78%–94% efficiency.	Storage at 4 ^∘^C.	Food product	[206]
*B. longum* BL-05	Extrusion	Alginate, pectin, and WPC	85.49%–95.21% efficiency	In simulated gastric juice for 2 h.	Effective delivery of probiotics	[207]
*L. acidophilus*	Encapsulation	Sodium alginate and carrageenan	Viability was improved.	120 days at −20 ^∘^C	Used in ice cream	[208]
*L. acidophilus* and *L. casei*	Freeze-drying	Whey protein isolate and fructooligosaccharides	Higher viability under simulated gastrointestinal fluid.	4 ^∘^C and 25 ^∘^C for 30 days	Food carriers	[83]
*L. casei* (PTCC 1608) and *B. coagulans* (GBI-30, 6086)	Encapsulation	Chitosan-gelatin films	B. coagulans was found more viable than L. casei.	4 ± 1 ^∘^C for 14 days	Chicken fillets preservation	[113]
*L. acidophilus* and *L. plantarum*	Encapsulation	Whey protein isolate and carrageenan	A 2–3 log_10_ CFU/g reduction occur in blended encapsulated cells.	4 ^∘^C and 25 ^∘^C for 30 days	Probiotic carriers	[180]
*L. plantarum* WCFS1	Electrodripping	Pectin and alginate or chitosan	Greater viability and less epithelial disruption.	-	Probiotic delivery	[141]
*L. acidophilus* LA-5 and *B. animalis* BB-12	Freeze-drying	Sodium alginate and pectin	Higher survivability in gut conditions.	4 ^∘^C for 30 days	To produce probiotic microcapsules	[209]
*L. acidophilus* La-05	Ionic gelation	Alginate and chitosan	Higher survivability during storage, thermal conditions, and gastric conditions.	7 ^∘^C for 120 days	Vegan milks as probiotic carriers	[210]
*L. acidophilus* NCIMB 701748	Spray-drying	Maltodextrin, whey protein concentrates, and D-glucose	Good storage stability and higher viability.	4 ^∘^C and 25 ^∘^C for 30 days	Probiotic formulations	[84]
*Lactiplantibacillus plantarum* NRRL B-4496 and *L*. *acidophilus* NRRL B-4495	Emulsification	Alginate	Stable for 4 months.	5 ^∘^C and 25 ^∘^C for 120 days	Food application	[80]
*L. paracasei*	Coaxial electrospinning	Starch-formate	Showed greater stability for 3 weeks.	4 ^∘^C for 21 days	Biotherapeutic encapsulation	[76]
*B. animalis* ssp. *lactis* (BB-12)	Spray-dried	Alginate, β-cyclodextrin, and xanthan gum	Improve the health of consumers and remain viable.	45 days	Functional food	[193]
*Lactobacillus rhamnosus* R011	Microentrapment	Whey protein isolate	Protection during heating, storage, and processing.	23 ^∘^C for 2 weeks in biscuit	Probiotic delivery vehicle	[191]

Abbreviations: ATCC: American Type Culture Collection; CFU: Colony-forming units; GG: Gorbach–Goldin; LAB: Lactic acid bacteria; NCIMB: National Collection of Industrial, Food and Marine Bacteria; NRRL: Northern Regional Research Laboratory (culture collection); PTCC: Persian Type Culture Collection; ssp.: Subspecies.

### Probiotication of food substances

Probiotication, the process of incorporating probiotics into food substances, offers substantial benefits, enhancing the nutritional value and health outcomes of food products. This process can be achieved through fermentation of food products or direct addition of probiotics [34]. Among the available techniques, microencapsulation has emerged as one of the most effective methods for integrating probiotics into various food matrices. Encapsulation provides several advantages, including improved cell viability, enhanced survivability during processing and storage, and protection against environmental stresses encountered during handling and transport. Furthermore, the addition of encapsulated probiotics contributes to the overall nutritional profile, health benefits, and consumer acceptance of functional foods [12, 34]. However, despite these benefits, challenges persist, such as maintaining sensory quality, ensuring controlled release, and achieving cost-effective large-scale production [12, 187].

### Bakery products

Probiotics play a significant role in the bakery industry, which contributes substantially to the food sector. Bakery products, including cakes, pastries, biscuits, and bread, are staples in daily diets. Collectively, the bakery and related industries bolster the economy. The incorporation of probiotics into bakery products can enhance health benefits and improve product quality [188, 189]. However, the addition of probiotics faces challenges, particularly during storage, packaging, cooking, and transportation [34].

To address these challenges, the encapsulation of probiotics presents a promising solution. Encapsulated materials protect probiotics and thereby enhance food quality. For example, the incorporation of *L. acidophilus* probiotic into bakery items was achieved using a starch edible film at high temperatures, yielding a survival rate of approximately 63% post-baking and 10% after 24 h of storage [34, 190]. Similarly, the introduction of *L. rhamnosus*R011 via microencapsulated whey protein into biscuits demonstrated a decrease of 2 log cycles after 24 h and 5 log cycles after three weeks of storage [191]. Furthermore, the inoculation of *L. reuteri* DSM 17938 via chitosan-alginate microcapsules in chocolate resulted in a 10% survival rate compared to just 1% without microencapsulation, following baking at 180 ^∘^C for 10 min [192]. Critical assessments of probiotic integration in bakery products indicate that, despite the protective effects of encapsulation, the final viable counts after baking often remain below the minimum therapeutic threshold recommended for probiotic functionality. Studies have indicated that survival rates can diminish during baking even with encapsulation, suggesting that current methods may not fully deliver the anticipated health benefits of probiotic-enriched bakery products. Therefore, further optimization of encapsulation materials, process parameters, and post-baking fortification strategies is essential to achieve the desired probiotic dosage in bakery matrices [190, 213].

### Meat-based substances

The incorporation of probiotics into meat-based products represents a promising strategy to enhance their functional and health-promoting properties. However, maintaining probiotic viability during processing, storage, and digestion is challenging due to environmental stresses such as heat, oxygen exposure, and low pH [214, 215]. To mitigate these issues, microencapsulation and immobilization have shown promising results. Research indicates that the incorporation of encapsulated probiotics in fermented or processed meats can improve microbial safety, oxidative stability, and shelf life, while also contributing to favorable flavor development [215]. Microencapsulated strains of *L. reuteri* ATCC 55730 and *B. longum* demonstrated higher viability during processing, fermentation, and drying when added to sausages [34]. The addition of alginate, β-cyclodextrin, and XG-based encapsulated *B. animalis ssp. lactis* (BB-12) to fermented meat products, such as Italian salami, resulted in greater viability (above 8 log CFU/g) for 45 days at 25 ^∘^C. Notably, this addition did not adversely affect other food properties, such as texture, nutritional value, or lipid profiling [193]. Conversely, some studies have indicated that encapsulated probiotics do not enhance the properties of probiotic-infused products. For instance, Camargo et al. [194] observed a decline in viability of encapsulated *B. animalis ssp. Lactis* (BB12) in meat (coppa), from 10.60 CFU/g to 7.3 log CFU/g, along with low lipid oxidation and increased weight loss. It is crucial to emphasize that the selection of encapsulation material must balance probiotic protection with the maintenance of food quality. While robust encapsulation materials provide superior protection, they may also alter the texture, flavor, or appearance of the final product. Therefore, optimizing the encapsulation matrix is vital to preserve both functional efficacy and sensory acceptability, ensuring the product appeals to consumers while delivering its intended health benefits [12, 215].

### Vegetable and fruit-based products

Probiotics are widely utilized in fruit- and vegetable-based products. The abundance and variety of these substances present a promising market for probiotic applications. Probiotics can be added in various forms to enhance the functionality and quality of these products [187, 195]. Studies have identified higher viability of *L. casei* DN-114 001, *L. rhamnosus* GG, and *L. paracasei* NFBC43338 in different fruit juices, whereas *L. salivarius* UCC118, *L. salivarius* UCC500, and *B. lactis* BB-12 exhibited lower viability [34]. Encapsulated probiotics demonstrated improved viability during storage in apple juice. One study found that microencapsulated *L. rhamnosus* GG, combined with resistant starch and whey protein isolate, retained greater cell viability [196]. Another research effort showed that sodium alginate-based encapsulated probiotic *E. faecium*in sour cherry juice exhibited higher viability after two months of storage [197]. Deshpande et al. (2022) reported that the colony count of probiotic cultures decreased from 3.0 × 10^9^ to 1.5 × 10^9^ CFU/mL when *L. bulgaricus*and *L. plantarum*were encapsulated in sweet orange juice. This reduction suggests that although encapsulation provides some degree of protection, specific physicochemical properties of the juice matrix may still adversely affect probiotic viability during storage [187, 216].

### Dairy-based products

The dairy industry significantly contributes to national economies and is a vital area for economic stability. Probiotics are extensively applied in various dairy products, such as milk, yogurt, and cheese, serving as vehicles for probiotic delivery. Various factors, including pH, salt concentration, and humidity, can influence probiotic effectiveness in dairy products, which encapsulation can help mitigate [34, 63]. Encapsulation has emerged as an effective strategy to enhance the stability, survivability, and functional performance of probiotics in dairy-based products. It improves microbial viability and can also influence the sensory and textural attributes of the food. Therefore, it is imperative to recognize that the choice of EM must strike a balance between effective probiotic protection and preservation of food quality. Generally, thick or incompatible coatings may compromise the sensory characteristics of the product, while insufficient protection can result in diminished probiotic efficacy. Thus, rational design of encapsulation systems is essential to ensure both probiotic functionality and consumer acceptance of the final dairy product [217, 218]. Research on the effectiveness of microencapsulated probiotics in yogurt indicated minimal reduction (0.07 log) in the viability of *B. infantis* 17930 and L. *rhamnosus* GG [198]. Mortazavian et al. (2008) utilized encapsulated *L. acidophilus* La-5 and *B. lactis* Bb-12 strains, observing their survival in yogurt drinks over six weeks, with encapsulated cells demonstrating a higher viability of 5.5 log cycles compared to 4.0 log cycles for free cells [217, 219].

### Other benefits

In addition to the previously mentioned advantages, encapsulated probiotics exhibit diverse applications across various sectors, particularly in non-dairy food matrices such as fruit juices, cereals, and plant-based beverages. Encapsulation enhances probiotic viability during processing and storage while protecting the cells from adverse physicochemical conditions typically encountered in these products, including low pH, oxygen exposure, and the presence of natural antimicrobial compounds [220, 221]. For instance, polyphenols in green tea, while recognized for their health benefits, may negatively impact probiotic cells by inhibiting their growth or metabolic activity during fermentation. To mitigate this issue, a whey protein-based encapsulated probiotic, *L. rhamnosus*, was utilized and stored. After 23 days at 4 ^∘^C, this strain demonstrated enhanced survivability [199]. Chewing gum, often categorized as junk food, presents a promising delivery vehicle for probiotics. Qaziyani et al. (2019) incorporated *Lactobacillus reuteri*, encapsulated in alginate, inulin, and lecithin, into chewing gum, concluding that the encapsulated strain remained viable after 21 days of storage. Furthermore, the inclusion of this strain improved the texture and sensory properties of the product [200].

### Role of encapsulated probiotics in biomedical and drug delivery systems

Beyond their applications in the food industry, encapsulated probiotics hold promise in biomedical and drug delivery systems due to their ability to endure harsh conditions. Encapsulation materials enhance their stress resistance and stability during manufacturing, storage, and transit through the GIT. This process not only preserves viability but also facilitates targeted delivery to specific intestinal sites [115, 164]. A critical determinant of successful targeted delivery is the formulation design, which requires optimization of polymer types, combinations, coating thickness, and processing parameters. The diverse microbial community in the large intestine enzymatically degrades polysaccharide coatings, serving as the primary mechanism for colon-targeted release. Consequently, encapsulated medications or probiotics are selectively released through microflora-mediated breakdown in the colonic region, where the concentration of fermentative bacteria is highest [222, 223]. Co-encapsulation strategies that combine probiotics with functional components (such as prebiotics and polyphenols) and advanced manufacturing methods (including microfluidics, 3D printing, and electrospinning) have demonstrated enhanced stability, targeted release, and functional persistence *in vivo*, indicating potential for intervention in chronic diseases like inflammatory bowel disease, diabetes, and colorectal cancer [224].

Studies in animal models have shown that encapsulated probiotics exhibit improved colonization and immunomodulatory effects compared to non-encapsulated cells. However, the transition to clinical practice remains limited, as human trials investigating site-targeted delivery, functional outcomes (beyond survival), and safety of encapsulated formats are scarce. Moreover, standardization of dosage, release kinetics, and matrix performance is lacking. Therefore, while biomedical prospects are encouraging, they should be regarded as emerging rather than established [225].

### Challenges, limitations, and future perspectives

Current literature indicates the potential and promising results of encapsulated probiotic strains in various formats, substances, and applications. Nevertheless, several challenges and limitations must be addressed. These challenges encompass the selection and optimization of suitable encapsulation materials and methods, process effectiveness, preservation of enclosed cells’ integrity, assurance of timely and site-specific probiotic release, and thorough elucidation of environmental factors. Another concern involves achieving optimal encapsulation efficiency without compromising the sensory, textural, and nutritional quality of the final food product. While encapsulation materials can effectively shield probiotics from adverse environmental stresses, their compatibility with the food matrix and consumer acceptability must be carefully assessed. Therefore, considerations should primarily focus on maintaining the texture, flavor, and nutritional value of the final product rather than solely on the probiotics themselves [15, 63]. Additionally, protecting probiotics from moisture, air, and temperature, as well as determining the negative effects during storage, processing, and delivery, pose significant challenges. The costs associated with encapsulation, scalability, and commercialization also remain barriers to widespread adoption. Furthermore, during administration, encapsulated probiotics encounter pre-existing bacteria, leading to competition for mucosal attachment and nutrients, which presents additional challenges [15, 40, 52, 60, 63, 211].

The science of probiotic encapsulation is rapidly evolving, utilizing various methods, materials, and combinatorial approaches to achieve more promising outcomes. Despite technological advancements, several limitations continue to obstruct the full realization of probiotic encapsulation’s potential. These include the unavailability of specific instruments, high costs, and risks of uncontrolled damage or death of probiotic cells. Allergies to certain EMs also pose a limitation. Furthermore, the uncontrolled effects of encapsulation methods and materials on probiotics require thorough consideration [15, 60, 70].

The future of encapsulation technologies in probiotics appears promising. Given the extensive applications of probiotics in food, pharmaceuticals, and personal care sectors, there is growing interest among researchers to identify novel strains with probiotic potential and to develop effective protection methods for existing strains. Future research in the following areas may help address current challenges and limitations, ultimately enhancing the efficacy, stability, and applicability of probiotics across diverse industries.

**a) Development of smart encapsulation materials**: Advancements should focus on creating stimuli-responsive materials that react to GIT stimuli such as changes in pH, temperature, and enzymatic activity.

**b) Development of a synbiotic system**: An integrative approach that combines probiotics and prebiotics into a synbiotic system with enhanced properties should be pursued.

**c) Enhancing shelf life without sensory alterations**: Research efforts should prioritize extending the shelf life of probiotic products while preserving the sensory attributes of the food matrix.

**d) Exploring understudied probiotic strains**: Current research predominantly focuses on well-characterized genera such as *Lactobacilli* and *Bifidobacterium*. Less-explored strains may possess equal or even superior probiotic properties.

**e) Advancing automation and production**: The integration of automated systems is essential for the scalable, consistent, and cost-effective production of encapsulated probiotics, including their protection and storage.

**f) Identifying specific biomarkers for efficacy**: Developing specific biomarkers or target substances is vital for assessing and tracking probiotic attachment, colonization, and functional effectiveness.

**g) Integrating genetic engineering tools**: Advanced genetic technologies, such as CRISPR-Cas systems, present promising opportunities to enhance the stability, functionality, and specificity of encapsulated probiotics.

**h) Integrating artificial intelligence tools**: AI-based tools, including machine learning, deep learning, and neural networks, can optimize encapsulation techniques, predict strain behavior, and personalize probiotic formulations.

**i) Engaging stakeholders in product development**: Incorporating perspectives from consumers, policymakers, and regulatory authorities is crucial for the successful development, acceptance, and regulation of encapsulated probiotic products.

**j) Addressing regulatory challenges**: The regulatory frameworks governing encapsulated probiotic products are complex and fragmented. Establishing clear guidelines and harmonized standards is essential to facilitate product development and market access.

## Conclusion

The global probiotic market is experiencing rapid expansion, leading to an increasing demand for products that offer optimal nutritional value, sensory quality, and extended shelf life. Probiotic encapsulation has emerged as a promising strategy to meet these demands by enhancing cell viability, stability, and safe storage during processing and gastrointestinal transit. However, the success of encapsulation should not be solely measured by higher survival rates; it must also address critical challenges in the food processing industry, such as cost-effectiveness, scalability, and compatibility with existing systems.

The practical application of this technology from laboratory settings to large-scale production necessitates a careful balance between probiotic protection and production feasibility. A variety of natural and synthetic EMs are currently utilized to safeguard probiotics under harsh processing and gastrointestinal conditions. Various physical and chemical encapsulation methods exist, each presenting distinct advantages and disadvantages based on the product matrix and desired release profile.

Encapsulation has demonstrated promising results, significantly improving the stability and functionality of probiotics in diverse food applications. Strains from the *Lactobacillus* and *Bifidobacterium* genera have been effectively encapsulated using single-layer systems, double coatings, or composite materials to optimize protection and controlled release.

Importantly, future research must extend beyond the mere quantification of viable cells to validate the therapeutic efficacy of encapsulated probiotics through functional assessments, including metabolic activity, adherence capabilities, and immunomodulatory potential within host systems. Such evidence-based validation will ensure that encapsulation technologies not only enhance probiotic survivability but also improve their biological functionality and health-promoting effects.

## References

[1] Mackowiak PA (2013). Recycling metchnikoff: probiotics, the intestinal microbiome and the quest for long life. Front Public Health.

[2] Podolsky SH (2012). Metchnikoff and the microbiome. Lancet.

[3] Hill C, Guarner F, Reid G, Gibson GR, Merenstein DJ, Pot B (2014). The international scientific association for probiotics and prebiotics consensus statement on the scope and appropriate use of the term probiotic. Nat Rev Gastroenterol Hepatol.

[4] Hussain A, Koser N, Aun SM, Siddiqui MF, Malik S, Ali SA (2024). Deciphering the role of probiotics in mental health: a systematic literature review of psychobiotics. Benef Microbes.

[5] Naidu AS, Bidlack WR, Clemens RA (1999). Probiotic spectra of lactic acid bacteria (LAB). Crit Rev Food Sci Nutr.

[6] Hussain A, Abid Ali S.

[7] Nagpal R, Kumar A, Kumar M, Behare PV, Jain S, Yadav H (2012). Probiotics, their health benefits and applications for developing healthier foods: a review. FEMS Microbiol Lett.

[8] Xu C, Gantumur MA, Sun J, Guo J, Ma J, Jiang Z (2024). Design of probiotic delivery systems for targeted release. Food Hydrocoll.

[9] Liang D, Wu F, Zhou D, Tan B, Chen T (2024). Commercial probiotic products in public health: current status and potential limitations. Crit Rev Food Sci Nutr.

[10] Hussain A, Arifa AN, Qureshi SR, Parveen S, Nisa UH, Ali SA.

[11] Afzaal M, Saeed F, Ateeq H, Imran A, Yasmin I, Shahid A (2022). Survivability of probiotics under hostile conditions as affected by prebiotic-based encapsulating materials. Int J Food Prop.

[12] Misra S, Pandey P, Dalbhagat CG, Mishra HN (2022). Emerging technologies and coating materials for improved probiotication in food products: a review. Food Bioprocess Technol.

[13] Kechagia M, Basoulis D, Konstantopoulou S, Dimitriadi D, Gyftopoulou K, Skarmoutsou N (2013). Health benefits of probiotics: a review. ISRN Nutr.

[14] Chugh B, Kamal-Eldin A (2020). Bioactive compounds produced by probiotics in food products. Curr Opin Food Sci.

[15] Sun Q, Yin S, He Y, Cao Y, Jiang C (2023). Biomaterials and encapsulation techniques for probiotics: current status and future prospects in biomedical applications. Nanomaterials.

[16] Xu Y, Yan X, Zheng H, Li J, Wu X, Xu J (2024). The application of encapsulation technology in the food industry: classifications, recent advances, and perspectives. Food Chem X.

[17] Strompfová V, Lauková A, Ouwehand AC (2004). Lactobacilli and enterococci—Potential probiotics for dogs. Folia Microbiol (Praha).

[18] Sanders ME (2008). Probiotics: definition, sources, selection, and uses. Clin Infect Dis.

[19] de Melo Pereira GV, de Oliveira Coelho B, Magalhães Júnior AI, Thomaz-Soccol V, Soccol CR (2018). How to select a probiotic? A review and update of methods and criteria. Biotechnol Adv.

[20] Liu L, Helal SE, Peng N (2023). CRISPR-cas-based engineering of probiotics. BioDesign Res.

[21] Ma J, Lyu Y, Liu X, Jia X, Cui F, Wu X (2022). Engineered probiotics. Microb Cell Fact.

[22] Hussain A, Akram S, Ahmad D, Rehman M, Ahmed A, Ali SA (2023). Molecular assessment and validation of the selected enterococcal strains as probiotics. Probiotics Antimicrob Proteins.

[23] Roobab U, Batool Z, Manzoor MF, Shabbir MA, Khan MR, Aadil RM (2020). Sources, formulations, advanced delivery and health benefits of probiotics. Curr Opin Food Sci.

[24] Ishibashi N, Yamazaki S (2001). Probiotics and safety. Am J Clin Nutr.

[25] Pradhan D, Mallappa RH, Grover S (2020). Comprehensive approaches for assessing the safety of probiotic bacteria. Food Control.

[26] Sarita B, Samadhan D, Hassan MZ, Kovaleva EG (2025). A comprehensive review of probiotics and human health-current prospective and applications. Front Microbiol.

[27] Hanchi H, Mottawea W, Sebei K, Hammami R (2018). The genus enterococcus: between probiotic potential and safety concerns—An update. Front Microbiol.

[28] Al-Fakhrany OM, Elekhnawy E (2024). Next-generation probiotics: the upcoming biotherapeutics. Mol Biol Rep.

[29] Abouelela ME, Helmy YA (2024). Next-generation probiotics as novel therapeutics for improving human health: current trends and future perspectives. Microorganisms.

[30] Kaźmierczak-Siedlecka K, Skonieczna-żydecka K, Hupp T, Duchnowska R, Marek-Trzonkowska N, Połom K (2022). Next-generation probiotics—Do they open new therapeutic strategies for cancer patients?. Gut Microbes.

[31] Reuben RC, Roy PC, Sarkar SL, Rubayet Ul Alam ASM, Jahid IK (2020). Characterization and evaluation of lactic acid bacteria from indigenous raw milk for potential probiotic properties. J Dairy Sci.

[32] Anwer M, Wei MQ (2024). Harnessing the power of probiotic strains in functional foods: nutritive, therapeutic, and next-generation challenges. Food Sci Biotechnol.

[33] Xu C, Ban Q, Wang W, Hou J, Jiang Z (2022). Novel nano-encapsulated probiotic agents: encapsulate materials, delivery, and encapsulation systems. J Control Release.

[34] De Prisco A, Mauriello G (2016). Probiotication of foods: a focus on microencapsulation tool. Trends Food Sci Technol.

[35] Huq T, Fraschini C, Khan A, Riedl B, Bouchard J, Lacroix M (2017). Alginate based nanocomposite for microencapsulation of probiotic: effect of cellulose nanocrystal (CNC) and lecithin. Carbohydr Polym.

[36] Wendel U (2021). Assessing viability and stress tolerance of probiotics—A review. Front Microbiol.

[37] Liu X, Mao B, Tang X, Zhang Q, Zhao J, Chen W (2025). Bacterial viability retention in probiotic foods: a review. Crit Rev Food Sci Nutr.

[38] Wang X, Hu J, Zhang H, Zhou P (2025). Probiotics encapsulated via biological macromolecule for neurological therapy and functional food: a review. Probiotics Antimicrob Proteins.

[39] Gheorghita R, Anchidin-Norocel L, Filip R, Dimian M, Covasa M (2021). Applications of biopolymers for drugs and probiotics delivery. Polymers (Basel).

[40] Yang S, Wei S, Wu Y, Fang Y, Deng Z, Xu J (2024). Encapsulation techniques, action mechanisms, and evaluation models of probiotics: recent advances and future prospects. Food Front.

[41] Safeer Abbas M, Afzaal M, Saeed F, Asghar A, Jianfeng L, Ahmad A (2023). Probiotic viability as affected by encapsulation materials: recent updates and perspectives. Int J Food Prop.

[42] Dangi P, Chaudhary N, Chaudhary V, Virdi AS, Kajla P, Khanna P (2023). Nanotechnology impacting probiotics and prebiotics: a paradigm shift in nutraceuticals technology. Int J Food Microbiol.

[43] Kadam O, Dalai S, Chauhan B, Guru RR, Mitra S, Raytekar N (2025). Nanobiotechnology unveils the power of probiotics: a comprehensive review on the synergistic role of probiotics and advanced nanotechnology in enhancing geriatric health. Cureus.

[44] Chen A, Gong Y, Wu S, Du Y, Liu Z, Jiang Y (2025). Navigating a challenging path: precision disease treatment with tailored oral nano-armor-probiotics. J Nanobiotechnol.

[45] Ghosh S, Sarkar B, Kaushik A, Mostafavi E (2022). Nanobiotechnological prospects of probiotic microflora: synthesis, mechanism, and applications. Sci Total Environ.

[46] Vijayaram S, Razafindralambo H, Sun YZ, Piccione G, Multisanti CR, Faggio C (2024). Synergistic interaction of nanoparticles and probiotic delivery: a review. J Fish Dis.

[47] Ramadevi S, Meenakshi S (2022). An epitome on encapsulation of probiotics. Arch Mater Sci Eng.

[48] Yang X, Wang C, Wang Q, Zhang Z, Nie W, Shang L (2023). Armored probiotics for oral delivery. Smart Med.

[49] Yao M, Xie J, Du H, McClements DJ, Xiao H, Li L (2020). Progress in microencapsulation of probiotics: a review. Compr Rev Food Sci Food Saf.

[50] Latif A, Shehzad A, Niazi S, Zahid A, Ashraf W, Iqbal MW (2023). Probiotics: mechanism of action, health benefits and their application in food industries. Front Microbiol.

[51] Sharma H, Sharma S, Bajwa J, Chugh R, Kumar D (2023). Polymeric carriers in probiotic delivery system. Carbohydr Polym Technol Appl.

[52] D’Amico V, Cavaliere M, Ivone M, Lacassia C, Celano G, Vacca M (2025). Microencapsulation of probiotics for enhanced stability and health benefits in dairy functional foods: a focus on pasta filata cheese. Pharmaceutics.

[53] Govender M, Choonara YE, Kumar P, du Toit LC, van Vuuren S, Pillay V (2013). A review of the advancements in probiotic delivery: conventional vs. non-conventional formulations for intestinal flora supplementation. AAPS PharmSciTech.

[54] Baral KC, Bajracharya R, Lee SH, Han HK (2021). Advancements in the pharmaceutical applications of probiotics: dosage forms and formulation technology. Int J Nanomed.

[55] Garcia-Brand AJ, Quezada V, Gonzalez-Melo C, Bolaños-Barbosa AD, Cruz JC, Reyes LH (2022). Novel developments on stimuli-responsive probiotic encapsulates: from smart hydrogels to nanostructured platforms. Fermentation.

[56] Rajam R, Subramanian P (2022). Encapsulation of probiotics: past, present and future. Beni-Suef Univ J Basic Appl Sci.

[57] De Prisco A, Maresca D, Ongeng D, Mauriello G (2015). Microencapsulation by vibrating technology of the probiotic strain Lactobacillus reuteri DSM 17938 to enhance its survival in foods and in gastrointestinal environment. LWT Food Sci Technol.

[58] Zhang T, Shang C, Du T, Zhuo J, Wang C, Li B (2023). Cytoprotection of probiotics by nanoencapsulation for advanced functions. Trends Food Sci Technol.

[59] Razavi S, Janfaza S, Tasnim N, Gibson DL, Hoorfar M (2021). Microencapsulating polymers for probiotics delivery systems: preparation, characterization, and applications. Food Hydrocoll.

[60] Singh S, Gupta R, Chawla S, Gauba P, Singh M, Tiwari RK (2022). Natural sources and encapsulating materials for probiotics delivery systems: Recent applications and challenges in functional food development. Front Nutr.

[61] Vijayaram S, Sinha R, Faggio C, Ringø E, Chou CC (2024). Biopolymer encapsulation for improved probiotic delivery: advancements and challenges. AIMS Microbiol.

[62] Priya AJ, Vijayalakshmi SP, Raichur AM (2011). Enhanced survival of probiotic lactobacillus acidophilus by encapsulation with nanostructured polyelectrolyte layers through layer-by-layer approach. J Agric Food Chem.

[63] Arratia-Quijada J, Nuño K, Ruíz-Santoyo V, Andrade-Espinoza BA (2024). Nano-encapsulation of probiotics: need and critical considerations to design new non-dairy probiotic products. J Funct Foods.

[64] Zhu Y, Wang Z, Bai L, Deng J, Zhou Q (2021). Biomaterial-based encapsulated probiotics for biomedical applications: current status and future perspectives. Mater Des.

[65] Lopes SA, Roque-Borda CA, Duarte JL, Di Filippo LD, Borges Cardoso VM, Pavan FR (2023). Delivery strategies of probiotics from nano- and microparticles: trends in the treatment of inflammatory bowel disease—An overview. Pharmaceutics.

[66] Afzaal M, Saeed F, Saeed M, Azam M, Hussain S, Mohamed AA (2020). Survival and stability of free and encapsulated probiotic bacteria under simulated gastrointestinal and thermal conditions. Int J Food Prop.

[67] Ningsih HS, Chen LG, Chung R, Chou YJ (2021). An Investigation on spray-granulated, macroporous, bioactive glass microspheres for a controlled drug delivery system. Materials (Basel).

[68] Kiran F, Afzaal M, Shahid H, Saeed F, Ahmad A, Ateeq H (2023). Effect of protein-based nanoencapsulation on viability of probiotic bacteria under hostile conditions. Int J Food Prop.

[69] Gutiérrez-Álzate K, Beltrán-Cotta LA, dos Santos Rekowsky BS, Cavalheiro CP, Pereira da Costa M (2024). Micro- and nanoencapsulation of probiotics: exploring their impact on animal-origin foods. ACS Food Sci Technol.

[70] Agriopoulou S, Tarapoulouzi M, Varzakas T, Jafari SM (2023). Application of encapsulation strategies for probiotics: from individual loading to co-encapsulation. Microorganisms.

[71] Centurion F, Basit AW, Liu J, Gaisford S, Rahim MA, Kalantar-Zadeh K (2021). Nanoencapsulation for probiotic delivery. ACS Nano.

[72] Franco RF, Jimenez PC (2025). Pharmacological applications of electrospun nanofibers loaded with bioactive natural compounds and extracts: a systematic review. Drugs Drug Candidates.

[73] Wei L, Zhou D, Kang X (2021). Electrospinning as a novel strategy for the encapsulation of living probiotics in polyvinyl alcohol/silk fibroin. Innov Food Sci Emerg Technol.

[74] Ma J, Xu C, Yu H, Feng Z, Yu W, Gu L (2021). Electro-encapsulation of probiotics in gum Arabic-pullulan blend nanofibres using electrospinning technology. Food Hydrocoll.

[75] Yang H, Wen P, Feng K, Zong MH, Lou WY, Wu H (2017). Encapsulation of fish oil in a coaxial electrospun nanofibrous mat and its properties. RSC Adv.

[76] Lancuški A, Abu Ammar A, Avrahami R, Vilensky R, Vasilyev G, Zussman E (2017). Design of starch-formate compound fibers as encapsulation platform for biotherapeutics. Carbohydr Polym.

[77] Heffernan SP, Kelly AL, Mulvihill DM, Lambrich U, Schuchmann HP (2011). Efficiency of a range of homogenisation technologies in the emulsification and stabilization of cream liqueurs. Innov Food Sci Emerg Technol.

[78] Afzal A, Afzaal M, Saeed F, Shah YA, Raza MA, Khan MH (2024). Milk protein based encapsulation of probiotics and other food material: comprehensive review. Int J Food Prop.

[79] Haji F, Cheon J, Baek J, Wang Q, Tam KC (2022). Application of Pickering emulsions in probiotic encapsulation—A review. Curr Res Food Sci.

[80] da Silva SÂD, Batista L da SP, Diniz DS, Nascimento SS da C, Morais NS, de Assis CF (2023). Microencapsulation of probiotics by oil-in-water emulsification technique improves cell viability under different storage conditions. Foods.

[81] Bhatta S, Stevanovic Janezic T, Ratti C (2020). Freeze-drying of plant-based foods. Foods.

[82] Raddatz GC, Poletto G, Deus C de, Codevilla CF, Cichoski AJ, Jacob-Lopes E (2020). Use of prebiotic sources to increase probiotic viability in pectin microparticles obtained by emulsification/internal gelation followed by freeze-drying. Food Res Int.

[83] Massounga Bora AF, Li X, Zhu Y, Du L (2019). Improved viability of microencapsulated probiotics in a freeze-dried banana powder during storage and under simulated gastrointestinal tract. Probiotics Antimicrob Proteins.

[84] Behboudi-Jobbehdar S, Soukoulis C, Yonekura L, Fisk I (2013). Optimization of spray-drying process conditions for the production of maximally viable microencapsulated L. acidophilus NCIMB 701748. Dry Technol.

[85] Bhagwat A, Bhushette P, Annapure US (2020). Spray drying studies of probiotic Enterococcus strains encapsulated with whey protein and maltodextrin. Beni-Suef Univ J Basic Appl Sci.

[86] Chávarri M, Marañón I, Ares R, Ibáñez FC, Marzo F, Villarán M del C (2010). Microencapsulation of a probiotic and prebiotic in alginate-chitosan capsules improves survival in simulated gastro-intestinal conditions. Int J Food Microbiol.

[87] Silva TM da, Barin JS, Lopes EJ, Cichoski AJ, Flores EM de M, Silva C de B da (2019). Development, characterization and viability study of probiotic microcapsules produced by complex coacervation followed by freeze-drying. Cienc Rural.

[88] Islam F, Amer Ali Y, Imran A, Afzaal M, Zahra SM, Fatima M (2023). Vegetable proteins as encapsulating agents: Recent updates and future perspectives. Food Sci Nutr.

[89] Minj S, Anand S (2020). Whey proteins and its derivatives: bioactivity, functionality, and current applications. Dairy.

[90] Feng K, Huangfu L, Liu C, Bonfili L, Xiang Q, Wu H (2023). Electrospinning and electrospraying: emerging techniques for probiotic stabilization and application. Polymers (Basel).

[91] Liu B, Hu J, Yao H, Zhang L, Liu H (2023). Improved viability of probiotics encapsulated by layer-by-layer assembly using zein nanoparticles and pectin. Food Hydrocoll.

[92] Li S, Fan L, Li S, Sun X, Di Q, Zhang H (2023). Validation of layer-by-layer coating as a procedure to enhance lactobacillus plantarum survival during in vitro digestion, storage, and fermentation. J Agric Food Chem.

[93] Rodrigues FJ, Cedran MF, Bicas JL, Sato HH (2020). Encapsulated probiotic cells: Relevant techniques, natural sources as encapsulating materials and food applications—A narrative review. Food Res Int.

[94] Guerra-Valle M, Petzold G, Orellana-Palma P (2022). Optimization of encapsulation by ionic gelation technique of cryoconcentrated solution: a response surface methodology and evaluation of physicochemical characteristics study. Polymers (Basel).

[95] Song LX, Bai L, Xu XM, He J, Pan SZ (2009). Inclusion complexation, encapsulation interaction and inclusion number in cyclodextrin chemistry. Coord Chem Rev.

[96] Figueiredo J de A, Silva CR de P, Souza Oliveira MF, Norcino LB, Campelo PH, Botrel DA (2022). Microencapsulation by spray chilling in the food industry: opportunities, challenges, and innovations. Trends Food Sci Technol.

[97] Zabihollahi N, Alizadeh A, Almasi H, Hanifian S, Hamishekar H (2020). Development and characterization of carboxymethyl cellulose based probiotic nanocomposite film containing cellulose nanofiber and inulin for chicken fillet shelf life extension. Int J Biol Macromol.

[98] Puscaselu RG, Lobiuc A, Dimian M, Covasa M (2020). Alginate: from food industry to biomedical applications and management of metabolic disorders. Polymers (Basel).

[99] Du H, Liu M, Yang X, Zhai G (2015). The design of pH-sensitive chitosan-based formulations for gastrointestinal delivery. Drug Discov Today.

[100] Voragen AGJ, Coenen GJ, Verhoef RP, Schols HA (2009). Pectin, a versatile polysaccharide present in plant cell walls. Struct Chem.

[101] Akin-Ajani OD, Okunlola A.

[102] Aires da Silva D, Cristine Melo Aires G, da Silva Pena R.

[103] BeMiller JN.

[104] Yang Y, Zhang J, Li C (2024). Delivery of probiotics with cellulose-based films and their food applications. Polymers (Basel).

[105] Shokri J, Adibki K.

[106] Compart J, Singh A, Fettke J, Apriyanto A (2023). Customizing starch properties: a review of starch modifications and their applications. Polymers (Basel).

[107] Montoya-Yepes DF, Jiménez-Rodríguez AA, Aldana-Porras AE, Velásquez-Holguin LF, Méndez-Arteaga JJ, Murillo-Arango W (2024). Starches in the encapsulation of plant active ingredients: state of the art and research trends. Polym Bull.

[108] Zang J, Yin Z, Ouyang H, Liu Y, Liu Z, Yin Z (2025). Advances in the preparation, applications, challenges, and future trends of polysaccharide-based gels as food-grade delivery systems for probiotics: a review. Compr Rev Food Sci Food Saf.

[109] Thomas N, Puluhulawa LE, Cindana Mo’o FR, Rusdin A, Gazzali AM, Budiman A (2024). Potential of pullulan-based polymeric nanoparticles for improving drug physicochemical properties and effectiveness. Polymers (Basel).

[110] Agrawal S, Budhwani D, Gurjar P, Telange D, Lambole V (2022). Pullulan based derivatives: synthesis, enhanced physicochemical properties, and applications. Drug Deliv.

[111] Díaz-Montes E (2021). Dextran: sources, structures, and properties. Polysaccharides.

[112] Yucel Falco C, Falkman P, Risbo J, Cárdenas M, Medronho B (2017). Chitosan-dextran sulfate hydrogels as a potential carrier for probiotics. Carbohydr Polym.

[113] Salimiraad S, Safaeian S, Basti AA, Khanjari A, Nadoushan RM (2022). Characterization of novel probiotic nanocomposite films based on nano chitosan/nano cellulose/gelatin for the preservation of fresh chicken fillets. LWT.

[114] Ramos M, Valdés A, Beltrán A, Garrigós M (2016). Gelatin-based films and coatings for food packaging applications. Coatings.

[115] Elzoghby AO, Abo El-Fotoh WS, Elgindy NA (2011). Casein-based formulations as promising controlled release drug delivery systems. J Control Release.

[116] Acuña-Avila PE, Cortes-Camargo S, Jiménez-Rosales A (2021). Properties of micro and nano casein capsules used to protect the active components: a review. Int J Food Prop.

[117] Liu G, An D, Li J, Deng S (2023). Zein-based nanoparticles: preparation, characterization, and pharmaceutical application. Front Pharmacol.

[118] Cheng C, Sun M, Wang L, Wang H, Li L, Yang Q (2024). Zein and soy polysaccharide encapsulation enhances probiotic viability and modulates gut microbiota. LWT.

[119] Nikam A, Sahoo PR, Musale S, Pagar RR, Paiva-Santos AC, Giram PS (2023). A Systematic overview of eudragit^®^ based copolymer for smart healthcare. Pharmaceutics.

[120] Nikam V (2011). Eudragit a versatile polymer: a review. Pharmacologyonline.

[121] Chakraborty S (2017). Carrageenan for encapsulation and immobilization of flavor, fragrance, probiotics, and enzymes: a review. J Carbohydr Chem.

[122] Sormin RBD, Masela A, Idris I (2019). Physicochemical properties of carrageenan originated from Lermatang Village, Southwest Maluku District. IOP Conf Ser Earth Environ Sci.

[123] Costa EM, Silva S, Pereira CF, Ribeiro AB, Casanova F, Freixo R (2023). Carboxymethyl cellulose as a food emulsifier: are its days numbered?. Polymers (Basel).

[124] Brondi M, Florencio C, Mattoso L, Ribeiro C, Farinas C (2022). Encapsulation of trichoderma harzianum with nanocellulose/carboxymethyl cellulose nanocomposite. Carbohydr Polym.

[125] Abdihaji M, Mirzaei Chegeni M, Hadizadeh A, Farrokhzad N, Kheradmand Z, Fakhrfatemi P (2023). Polyvinyl alcohol (PVA)-based nanoniosome for enhanced in vitro delivery and anticancer activity of thymol. Int J Nanomedicine.

[126] Luo Y, De Souza C, Ramachandran M, Wang S, Yi H, Ma Z (2022). Precise oral delivery systems for probiotics: a review. J Control Release.

[127] Wang X, Gao S, Yun S, Zhang M, Peng L, Li Y (2022). Microencapsulating alginate-based polymers for probiotics delivery systems and their application. Pharmaceuticals.

[128] Wang Y, Lu Y (2023). Sodium alginate-based functional materials toward sustainable applications: water treatment and energy storage. Ind Eng Chem Res.

[129] Cao L, Lu W, Mata A, Nishinari K, Fang Y (2020). Egg-box model-based gelation of alginate and pectin: a review. Carbohydr Polym.

[130] Phùng TTT, Dinh HN, Ureña M, Oliete B, Denimal E, Dupont S (2025). Sodium alginate as a promising encapsulating material for extremely-oxygen sensitive probiotics. Food Hydrocoll.

[131] Bevilacqua A, Campaniello D, Speranza B, Racioppo A, Altieri C, Sinigaglia M (2020). Microencapsulation of saccharomyces cerevisiae into alginate beads: a focus on functional properties of released cells. Foods.

[132] Vega-Carranza AS, Cervantes-Chávez JA, Luna-Bárcenas G, Luna-González A, Diarte-Plata G, Nava-Mendoza R (2021). Alginate microcapsules as delivery and protective systems of Bacillus licheniformis in a simulated shrimp’s digestive tract. Aquaculture.

[133] Aranaz I, Alcántara AR, Civera MC, Arias C, Elorza B, Heras Caballero A (2021). Chitosan: an overview of its properties and applications. Polymers (Basel).

[134] Meyer-Déru L, David G, Auvergne R (2022). Chitosan chemistry review for living organisms encapsulation. Carbohydr Polym.

[135] Senthil Kumar S, Sheik Mohideen S (2025). Encapsulation of L. fermentum with chitosan-alginate enhances its bioactivity against acrylamide toxicity in D. mel. Sci Rep.

[136] Mohammed M, Syeda J, Wasan K, Wasan E (2017). An overview of chitosan nanoparticles and its application in non-parenteral drug delivery. Pharmaceutics.

[137] Wang X, Yu M, Ye J, Liu T, Jian L, Zheng X (2024). Research advances on encapsulation of probiotics with nanomaterials and their repair mechanisms on intestinal barriers. Food Sci Hum Wellness.

[138] Khosravi Zanjani MA, Ghiassi Tarzi B, Sharifan A, Mohammadi N (2014). Microencapsulation of probiotics by calcium alginate-gelatinized starch with chitosan coating and evaluation of survival in simulated human gastro-intestinal condition. Iran J Pharm Res.

[139] Peñalva R, Martínez-López AL, Gamazo C, Gonzalez-Navarro CJ, González-Ferrero C, Virto-Resano R (2023). Encapsulation of lactobacillus plantarum in casein-chitosan microparticles facilitates the arrival to the colon and develops an immunomodulatory effect. Food Hydrocoll.

[140] Chandel V, Biswas D, Roy S, Vaidya D, Verma A, Gupta A (2022). Current advancements in pectin: extraction, properties and multifunctional applications. Foods.

[141] Galvez-Jiron F, Tang X, Gasaly N, Poncelet D, Wandersleben T, Drusch S (2025). Pectin-based encapsulation systems for the protection of beneficial bacterial species and impact on intestinal barrier function in vitro. Food Hydrocoll.

[142] Bordini FW, Rosolen MD, da Luz G de Q, Pohndorf RS, de Oliveira PD, Conceição FR (2023). Development of a microencapsulated probiotic delivery system with whey, xanthan, and pectin. Braz J Microbiol.

[143] Butte K, Momin M, Deshmukh H (2014). Optimisation and in vivo evaluation of pectin based drug delivery system containing curcumin for colon. Int J Biomater.

[144] Li M, Jin Y, Wang Y, Meng L, Zhang N, Sun Y (2019). Preparation of Bifidobacterium breve encapsulated in low methoxyl pectin beads and its effects on yogurt quality. J Dairy Sci.

[145] Robyt JF.

[146] Zhou D, Li D, Liu M, Zhong X, Wei H, Wang Z (2021). Experimental parameters affecting cross-linking density and free-thaw stability of cross-linked porous starch. ES Food Agrofor.

[147] Noman M, Afzaal M, Saeed F, Ahmad A, Imran A, Akram N (2023). Effect of starch-based nanoparticles on the viability and stability of probiotics under adverse conditions. Int J Food Prop.

[148] Wang B, Sun X, Xu M, Wang F, Liu W, Wu B (2023). Structural characterization and partial properties of dextran produced by Leuconostoc mesenteroides RSG7 from pepino. Front Microbiol.

[149] Albadran HA, Monteagudo-Mera A, Khutoryanskiy VV, Charalampopoulos D (2020). Development of chitosan-coated agar-gelatin particles for probiotic delivery and targeted release in the gastrointestinal tract. Appl Microbiol Biotechnol.

[150] Sabio L, González A, Ramírez-Rodríguez GB, Gutiérrez-Fernández J, Bañuelo O, Olivares M (2021). Probiotic cellulose: antibiotic-free biomaterials with enhanced antibacterial activity. Acta Biomater.

[151] Aquinas N, Chithra CH, Bhat MR (2024). Progress in bioproduction, characterization and applications of pullulan: a review. Polym Bull.

[152] Elangwe CN, Morozkina SN, Olekhnovich RO, Polyakova VO, Krasichkov A, Yablonskiy PK (2023). Pullulan-based hydrogels in wound healing and skin tissue engineering applications: a review. Int J Mol Sci.

[153] Ajalloueian F, Guerra PR, Bahl MI, Torp AM, Hwu ETe, Licht TR (2022). Multi-layer PLGA-pullulan-PLGA electrospun nanofibers for probiotic delivery. Food Hydrocoll.

[154] Kycia K, Chlebowska-Śmigiel A, Szydłowska A, Sokół E, Ziarno M, Gniewosz M (2020). Pullulan as a potential enhancer of Lactobacillus and Bifidobacterium viability in synbiotic low fat yoghurt and its sensory quality. LWT.

[155] Hamdani AM, Wani IA, Bhat NA (2019). Sources, structure, properties and health benefits of plant gums: a review. Int J Biol Macromol.

[156] Barak S, Mudgil D, Taneja S (2020). Exudate gums: chemistry, properties and food applications—A review. J Sci Food Agric.

[157] Saeed M, Khanam R, Hafeez H, Ahmad Z, Saleem S, Tariq MR (2024). Viability of free and alginate-carrageenan gum coated lactobacillus acidophilus and lacticaseibacillus casei in functional cottage cheese. ACS Omega.

[158] Pandey S, Senthilguru K, Uvanesh K, Sagiri SS, Behera B, Babu N (2016). Natural gum modified emulsion gel as single carrier for the oral delivery of probiotic-drug combination. Int J Biol Macromol.

[159] Lu Y, Luo Q, Chu Y, Tao N, Deng S, Wang L (2022). Application of gelatin in food packaging: a review. Polymers (Basel).

[160] Abdel-Aty AM, Barakat AZ, Bassuiny RI, Mohamed SA (2024). Chia gum-gelatin-based encapsulation of chia sprouts phenolic compounds enhanced storage stability, bioavailability, antioxidant, antidiabetic, and antibacterial properties. Sci Rep.

[161] Hadidi M, Majidiyan N, Jelyani AZ, Moreno A, Hadian Z, Mousavi Khanegah A (2021). Alginate/fish gelatin-encapsulated lactobacillus acidophilus: a study on viability and technological quality of bread during baking and storage. Foods.

[162] Patarroyo JL, Florez-Rojas JS, Pradilla D, Valderrama-Rincón JD, Cruz JC, Reyes LH (2020). Formulation and characterization of gelatin-based hydrogels for the encapsulation of Kluyveromyces lactis—Applications in packed-bed reactors and probiotics delivery in humans. Polymers (Basel).

[163] Rama GR, Dullius D, Agnol WD, Esquerdo VM, Lehn DN, de Souza CFV (2021). Ricotta whey supplemented with gelatin and collagen for the encapsulation of probiotic lactic acid bacteria. Food Sci Technol.

[164] Gandhi S, Roy I (2021). Drug delivery applications of casein nanostructures: a minireview. J Drug Deliv Sci Technol.

[165] Głab TK, Boratyński J (2017). Potential of casein as a carrier for biologically active agents. Top Curr Chem.

[166] Abd El-Salam MH, El-Shibiny S (2015). Preparation and properties of milk proteins-based encapsulated probiotics: a review. Dairy Sci Technol.

[167] Yiğit A, Bielska P, Cais-Sokolińska D, Samur G (2023). Whey proteins as a functional food: health effects, functional properties, and applications in food. J Am Nutr Assoc.

[168] Jiang L, Zhang Z, Qiu C, Wen J (2024). A review of whey protein-based bioactive delivery systems: design, fabrication, and application. Foods.

[169] Le NTM, Hieu N Van (2018). Use of whey protein for encapsulation and controlled release of probiotic from protein microencapsule in ex vivo porcine gastrointestinal contents. Vietnam J Sci Technol.

[170] de Araújo Etchepare M, Nunes GL, Nicoloso BR, Barin JS, Moraes Flores EM, de Oliveira Mello R (2020). Improvement of the viability of encapsulated probiotics using whey proteins. LWT.

[171] Diep Huy Vũ P, Rodklongtan A, Chitprasert P (2021). Whey protein isolate-lignin complexes as encapsulating agents for enhanced survival during spray drying, storage, and in vitro gastrointestinal passage of Lactobacillus reuteri KUB-AC5. LWT.

[172] Oleandro E, Stanzione M, Buonocore GG, Lavorgna M (2024). Zein-based nanoparticles as active platforms for sustainable applications: recent advances and perspectives. Nanomaterials.

[173] Shanthakumar P, Klepacka J, Bains A, Chawla P, Dhull SB, Najda A (2022). The current situation of pea protein and its application in the food industry. Molecules.

[174] Babot JD, Argañaraz-Martínez E, Apella MC, Perez Chaia A (2023). Microencapsulation of probiotics with soy protein isolate and alginate for the poultry industry. Food Bioprocess Technol.

[175] Akanny E, Bourgeois S, Bonhommé A, Commun C, Doleans-Jordheim A, Bessueille F (2020). Development of enteric polymer-based microspheres by spray-drying for colonic delivery of Lactobacillus rhamnosus GG. Int J Pharm.

[176] Ansari F, Pourjafar H, Jodat V, Sahebi J, Ataei A (2017). Effect of Eudragit S100 nanoparticles and alginate chitosan encapsulation on the viability of Lactobacillus acidophilus and Lactobacillus rhamnosus. AMB Express.

[177] Rahmati F (2019). Impact of microencapsulation on two probiotic strains in alginate chitosan and eudragit S100 under gastrointestinal and normal conditions. Open Biotechnol J.

[178] Li Z, Cheong KL, Song B, Yin H, Li Q, Chen J (2024). Preparation of κ-carrageenan oligosaccharides by photocatalytic degradation: Structural characterization and antioxidant activity. Food Chem X.

[179] Álvarez-Viñas M, Souto S, Flórez-Fernández N, Torres MD, Bandín I, Domínguez H (2021). Antiviral activity of carrageenans and processing implications. Mar Drugs.

[180] Sogut E, Filiz BE, Seydim AC (2022). Whey protein isolate- and carrageenan-based edible films as carriers of different probiotic bacteria. J Dairy Sci.

[181] Subaşı-Zarbaliyev B, Kutlu G, Törnük F (2023). Polyvinyl alcohol nanoparticles loaded with propolis extract: fabrication, characterization and antimicrobial activity. ADMET DMPK.

[182] Corona-Escalera AF, Tinajero-Díaz E, García-Reyes RA, Luna-Bárcenas G, Seyfoddin A, Padilla-de la Rosa JD (2022). Enzymatic crosslinked hydrogels of gelatin and poly (vinyl alcohol) loaded with probiotic bacteria as oral delivery system. Pharmaceutics.

[183] Çanga EM, Dudak FC (2021). Improved digestive stability of probiotics encapsulated within poly(vinyl alcohol)/cellulose acetate hybrid fibers. Carbohydr Polym.

[184] Mojaveri SJ, Hosseini SF, Gharsallaoui A (2020). Viability improvement of Bifidobacterium animalis Bb12 by encapsulation in chitosan/poly(vinyl alcohol) hybrid electrospun fiber mats. Carbohydr Polym.

[185] Thabet OA, Al Muzini FS, Atiya AM, Alamry KA, Hussein MA, Hoogenboom R (2023). Hydrophobic carboxymethyl cellulose as a clean-up sorbent in the determination of nitrofuran metabolites in animal-fat samples. RSC Adv.

[186] Yousefi N, Zahedi Y, Yousefi A, Hosseinzadeh G, Jekle M (2024). Development of carboxymethyl cellulose-based nanocomposite incorporated with ZnO nanoparticles synthesized by cress seed mucilage as green surfactant. Int J Biol Macromol.

[187] Rahman M, Emon D, Toma M, Nupur A, Karmoker P, Iqbal A (2023). Recent advances in probiotication of fruit and vegetable juices. J Adv Vet Anim Res.

[188] Pejcz E (2024). Biotechnological approach of technological advancements for sustainable probiotic bread production. Sustainability.

[189] Sadeghi A, Ebrahimi M, Assadpour E, Jafari SM (2024). Recent advances in probiotic breads; a market trend in the functional bakery products. Crit Rev Food Sci Nutr.

[190] Altamirano-Fortoul R, Moreno-Terrazas R, Quezada-Gallo A, Rosell CM (2012). Viability of some probiotic coatings in bread and its effect on the crust mechanical properties. Food Hydrocoll.

[191] Reid AA, Champagne CP, Gardner N, Fustier P, Vuillemard JC (2007). Survival in food systems of lactobacillus rhamnosus R011 microentrapped in whey protein gel particles. J Food Sci.

[192] Malmo C, La Storia A, Mauriello G (2013). Microencapsulation of lactobacillus reuteri DSM 17938 cells coated in alginate beads with chitosan by spray drying to use as a probiotic cell in a chocolate soufflé. Food Bioprocess Technol.

[193] de Oliveira Gomes B, de Mesquita Oliveira C, de Marins AR, Gomes RG, Feihrmann AC (2021). Application of microencapsulated probiotic Bifidobacterium animalis ssp. lactis BB-12 in Italian salami. J Food Process Preserv.

[194] Camargo VP, Catanio N, Marins AR de, Bergamasco R de C, Gomes RG, Feihrmann AC (2021). The physicochemical and sensory characteristics of coppa with bifidobacterium animalis ssp. lactis (BB12) probiotic. Acta Sci Technol.

[195] Hussain A, Parveen S, Riaz M, Zia A, Abid Ali S (2024). Probiotics and vegetable oil association: a review. IOP Conf Ser Earth Environ Sci.

[196] Ying D, Schwander S, Weerakkody R, Sanguansri L, Gantenbein-Demarchi C, Augustin MA (2013). Microencapsulated Lactobacillus rhamnosus GG in whey protein and resistant starch matrices: probiotic survival in fruit juice. J Funct Foods.

[197] Azarkhavarani PR, Ziaee E, Hosseini SMH (2019). Effect of encapsulation on the stability and survivability of enterococcus faecium in a non-dairy probiotic beverage. Food Sci Technol Int.

[198] Capela P, Hay TKC, Shah NP (2006). Effect of cryoprotectants, prebiotics and microencapsulation on survival of probiotic organisms in yoghurt and freeze-dried yoghurt. Food Res Int.

[199] Hernández-Barrueta T, Martínez-Bustos F, Castaño-Tostado E, Lee Y, Miller MJ, Amaya-Llano SL (2020). Encapsulation of probiotics in whey protein isolate and modified huauzontle’s starch: an approach to avoid fermentation and stabilize polyphenol compounds in a ready-to-drink probiotic green tea. LWT.

[200] Qaziyani SD, Pourfarzad A, Gheibi S, Nasiraie LR (2019). Effect of encapsulation and wall material on the probiotic survival and physicochemical properties of synbiotic chewing gum: study with univariate and multivariate analyses. Heliyon.

[201] Dafe A, Etemadi H, Zarredar H, Mahdavinia GR (2017). Development of novel carboxymethyl cellulose/κ-carrageenan blends as an enteric delivery vehicle for probiotic bacteria. Int J Biol Macromol.

[202] Su R, Zhu XL, Fan DD, Mi Y, Yang CY, Jia X (2011). Encapsulation of probiotic Bifidobacterium longum BIOMA 5920 with alginate-human-like collagen and evaluation of survival in simulated gastrointestinal conditions. Int J Biol Macromol.

[203] Fareez IM, Lim SM, Mishra RK, Ramasamy K (2015). Chitosan coated alginate-xanthan gum bead enhanced pH and thermotolerance of Lactobacillus plantarum LAB12. Int J Biol Macromol.

[204] Khorasani AC, Shojaosadati SA (2016). Bacterial nanocellulose-pectin bionanocomposites as prebiotics against drying and gastrointestinal condition. Int J Biol Macromol.

[205] Bu W, McClements DJ, Zhang Z, Zhang R, Jin Z, Chen L (2025). Encapsulation method of probiotic embedded delivery system and its application in food. Food Hydrocoll.

[206] Peredo AG, Beristain CI, Pascual LA, Azuara E, Jimenez M (2016). The effect of prebiotics on the viability of encapsulated probiotic bacteria. LWT.

[207] Yasmin I, Saeed M, Pasha I, Zia MA (2019). Development of whey protein concentrate-pectin-alginate based delivery system to improve survival of B. longum BL-05 in simulated gastrointestinal conditions. Probiotics Antimicrob Proteins.

[208] Afzaal M, Saeed F, Arshad MU, Nadeem MT, Saeed M, Tufail T (2019). The effect of encapsulation on the stability of probiotic bacteria in ice cream and simulated gastrointestinal conditions. Probiotics Antimicrob Proteins.

[209] Motalebi Moghanjougi Z, Rezazadeh Bari M, Alizadeh Khaledabad M, Amiri S, Almasi H (2021). Microencapsulation of Lactobacillus acidophilus LA-5 and Bifidobacterium animalis BB-12 in pectin and sodium alginate: a comparative study on viability, stability, and structure. Food Sci Nutr.

[210] Andrade Lopes LA, de Siqueira Ferraz Carvalho R, Santos Magalhães N, Madruga MS, Alves Aguiar Athayde AJ, Araújo Portela I (2020). Microencapsulation of Lactobacillus acidophilus La-05 and incorporation in vegan milks: physicochemical characteristics and survival during storage, exposure to stress conditions, and simulated gastrointestinal digestion. Food Res Int.

[211] Rokka S, Rantamäki P (2010). Protecting probiotic bacteria by microencapsulation: challenges for industrial applications. Eur Food Res Technol.

[212] Saiz-Gonzalo G, Arroyo-Moreno S, McSweeney S, Bleiel SB (2025). Pea protein microencapsulation improves probiotic survival during gastrointestinal digestion. Int J Food Sci Technol.

[213] Beikzadeh S, Sadeghi A, Khezerlou A, Assadpour E (2025). Enrichment of bread with encapsulated probiotics as a functional product containing bioactive compounds: principles, outcomes, and challenges. Future Foods.

[214] Arihara K (2006). Strategies for designing novel functional meat products. Meat Sci.

[215] Munekata PES, Pateiro M, Tomasevic I, Domínguez R, da Silva Barretto AC, Santos EM (2022). Functional fermented meat products with probiotics—A review. J Appl Microbiol.

[216] Deshpande HW, Katke SD, Poshadri A (2022). Influence of probiotics on physico-chemical and organoleptic characteristics of sweet orange juice. J Environ Biol.

[217] Vivek KB (2013). Use of encapsulated probiotics in dairy based foods. Int J Food Agric Vet Sci.

[218] Iravani S, Korbekandi H, Mirmohammadi SV (2015). Technology and potential applications of probiotic encapsulation in fermented milk products. J Food Sci Technol.

[219] Mortazavian AM, Ehsani MR, Azizi A, Razavi SH, Mousavi SM, Sohrabvandi S (2008). Viability of calcium-alginate microencapsulated probiotic bacteria in Iranian yogurt drink (Doogh) during refrigerated storage and under simulated gastrointestinal conditions. Aust J Dairy Technol.

[220] Küçükgöz K, Trzaskowska M (2022). Nondairy probiotic products: functional foods that require more attention. Nutrients.

[221] Kumar S, Rattu G, Mitharwal S, Chandra A, Kumar S, Kaushik A (2022). Trends in non-dairy-based probiotic food products: advances and challenges. J Food Process Preserv.

[222] Yoha KS, Nida S, Dutta S, Moses JA, Anandharamakrishnan C (2022). Targeted delivery of probiotics: perspectives on research and commercialization. Probiotics Antimicrob Proteins.

[223] Ibrahim IM (2023). Advances in polysaccharide-based oral colon-targeted delivery systems: the journey so far and the road ahead. Cureus.

[224] Ji C, Li D, Liang Y, Luo Y (2025). Co-encapsulation of probiotics with functional components: design strategies, synergistic mechanisms, biomedical applications, and challenges for industrialization. J Mater Chem B.

[225] Razavi S, Janfaza S, Tasnim N, Gibson DL, Hoorfar M (2021). Nanomaterial-based encapsulation for controlled gastrointestinal delivery of viable probiotic bacteria. Nanoscale Adv.

